# Precise Tailoring 3D Printed In Situ Toughening Low‐k Microwave Transparent Structure via Thiol‐acrylate Chain Transfer Behavior

**DOI:** 10.1002/advs.74759

**Published:** 2026-03-10

**Authors:** Ce Bian, Kai Zheng, Ruoyu Chen, Wansong Gu, Guanxiang Zhong, Difei Liang, Longjiang Deng, Hetao Chu

**Affiliations:** ^1^ National Engineering Research Center of Electromagnetic Radiation Control Materials University of Electronic Science and Technology of China (UESTC) Chengdu China; ^2^ Key Laboratory of Multispectral Absorbing Materials and Structures of Ministry of Education University of Electronic Science and Technology of China (UESTC) Chengdu China

**Keywords:** Low‐*k* materials, thiol‐acrylate chain transfer, microwave transparency, DLP 3D printing

## Abstract

The unique electromagnetic properties of triazine resins have enabled their growing use in advanced low‐dielectric (low‐*k*) materials. However, their low printability and high brittleness severely restrict engineering applications. Conventional plasticization strategies that enhance printability often compromise dielectric and mechanical properties. Herein, an in situ thiol–acrylate chain transfer‐mediated toughening strategy is introduced for Digital Light Processing (DLP) printing. A triazine‐based resin system comprising THEICTA, isobornyl acrylate (IBOA), and polysulfide rubber (PSR) is formulated into a low‐viscosity (η < 150 cP), highly photosensitive ink, enabling 50 µm resolution printing while preserving a low dielectric constant. Compared with the homopolymer of triazine resin, the topology‐optimized molecular network simultaneously delivers a 462.4% increase in elongation at break, a 640.5% improvement in toughness, a high tensile strength (45.7 MPa), and a low dielectric constant (*ε′* = 2.66). Benefiting from its excellent printability, a printed gyroid lattice‐filled plate (100 µm wall thickness) achieves 92% transmittance at 10 GHz under a 50° incidence angle following structural optimization.

## Graphical abstract

An in situ thiol–acrylate chain transfer‐mediated toughening strategy is introduced for Digital Light Processing (DLP) printing. Benefiting from its excellent printability, a printed gyroid lattice‐filled plate (100 µm wall thickness) achieves 92% transmittance at 10 GHz under a 50° incidence angle following structural optimization.

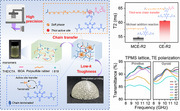

## 1 Introduction

The advancements in radar systems have heightened the demand for faster signal transmission and higher signal quality. Low dielectric constant materials play a crucial role in minimizing delay and signal loss during transmission, thereby supporting next‐generation communications [1, 2, 3, 4]. Meanwhile, the additive manufacturing technologies have made significant strides, offering versatile fabrication routes for complex geometries and lightweight structures. Among these, DLP technology stands out for its exceptional precision, printing speed, and scalability [5, 6, 7]. The synergy between low‐*k* materials and additive manufacturing holds considerable promise for applications in communication engineering, aerospace, and integrated circuit fields.

Triazine rings (six‐membered aromatic heterocycles containing three nitrogen atoms) are often used in high‐performance materials such as cyanate ester resins and polybenzotriazines [8]. Among them, photocurable triazine resins have attracted considerable attention due to their potential printability, but their inherently solid‐state and brittleness pose significant challenges for high‐resolution DLP printing. Therefore, it is essential to design the resin formulations to mitigate brittleness while maintaining the low dielectric constant and mechanical properties. Typically, reactive diluents are often incorporated to modify rheological behaviors and achieve suitable viscosity for DLP printing [9, 10, 11]. Conventional strategies to improve the toughness of photocurable resins involve the incorporation of inorganic nanoparticles, rubbers, thermoplastics, and thermosets [12, 13, 14, 15]. However, these methods exhibit limitations in balancing mechanical and dielectric properties. Inorganic nanofillers tend to agglomerate in the printing ink system, compromising dispersion uniformity and leading to low printing resolution [16]. Rubber modifiers usually exhibit poor interfacial bonding with photocurable monomers, due to the lack of reactive functional groups [17]. Traditional thermosetting resins generally possess a higher dielectric constant and dielectric loss than triazine‐based systems, making it difficult to meet the performance requirements of low‐*k* wave‐transparent structures [18, 19].

For the thiol‐acrylate system, it has been demonstrated that thiol functional groups can undergo chain transfer reactions with unsaturated double bonds and photoinitiator during free radical polymerization. Cramer and Bowman reported that the introduction of thiols transforms the polymerization mechanism into a binary system [20], in which the network formation proceeds through both chain‐growth and step‐growth processes. Previous studies have demonstrated that the variation of polymerization mechanism can markedly affect the topological structure of polymer network, thereby enabling tailoring of the mechanical and dielectric properties of crosslinked materials [21]. For instance, Thijssen et al. employed a tetrafunctional thiol as a chain transfer agent to regulate the network topology of polycaprolactone (PCL) composites [22], achieving notable improvements in elongation at break (707 ± 101%), ultimate tensile strength (13.9 ± 1.5 MPa), and toughness. Tayler S. Hebner et al. constructed liquid crystal elastomer (LCE) polymer networks via the photopolymerization of diacrylate liquid crystal monomers with dithiols [23], and optimized the mechanical properties by adjusting the ratio of chain transfer agents. To date, most studies have focused on utilizing small‐molecule thiols to enhance photocurable elastomers or gel‐like materials. However, thiol‐acrylate chain transfer has rarely been exploited as a topology‐regulation tool in high printing resolution, in situ mechanical toughening, and low‐*k* 3D printing photocurable systems.

In this study, we employ thiol‐terminated liquid polysulfide rubber (JLY‐121, PSR) as an in situ toughening agent, which polymerizes directly within a triazine‐based matrix [24, 25, 26], enhancing overall performance. Through the effect of thiol‐acrylate chain transfer behavior, we achieve high printing resolution, in situ toughening, and low‐*k* properties. The chosen triazine resin, tris(*2‐*hydroxyethyl) isocyanurate triacrylate (THEICTA) featured trifunctional acrylate groups capable of rapidly curing with high glass transition temperature (*T_g_* 247°C) [27], low‐*k* (*ε′* 3.03–3.06, X‐band), and superior mechanical strength networks. A low‐viscosity monomer, isobornyl acrylate (IBOA), is enrolled as a reactive diluent to reduce viscosity and facilitate processing. The results demonstrate that the chain transfer mechanism regulates and controls the network topology, reducing crosslinking density and increasing free volume. Furthermore, a series of triply periodic minimal surface (TPMS) lattices is fabricated [28]. The lightweight structures exhibit excellent printing resolution, wave‐transparency, and mechanical strength, which is appropriate for the structural design of curved radomes. In summary, this work applies an in situ thiol–acrylate chain transfer mediated toughening strategy that achieves gains in both mechanical and dielectric properties. Notably, the dielectric constant achieved at GHz frequencies compares favorably with representative reported photopolymers, as summarized in Table S1. These findings provide a viable molecular and structural design strategy for high‐performance low‐k printable materials.

## 2 Results and Discussion

### 2.1 Ink Preparation and Polymer Network Construction

**FIGURE 1:**
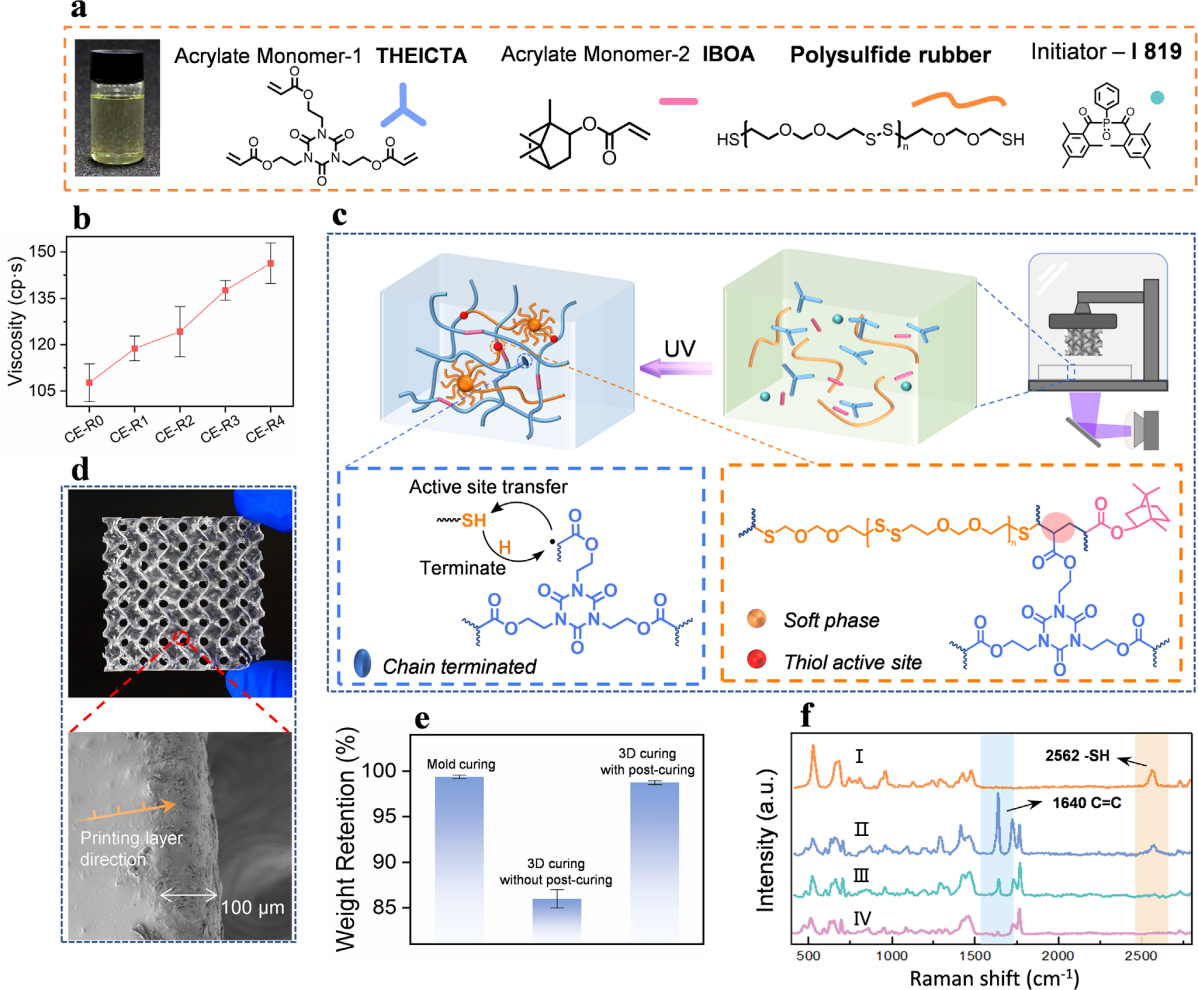
(a) Composition and chemical structure of 3D printing ink. (b) The viscosity of ink with different content of PSR. (c) The scheme of DLP 3D printing and the mechanism of Thiol‐acrylate chain transfer. (d) The optical and SEM images display printing continuity and accuracy of CE‐R2 model. (e) The weight retention ratio of different processes. (f) Raman spectra of (I) PSR, (II) inks before 3D printing, (III) inks after 3D printing, and (IV) inks after post‐curing.

Photocurable resins exhibiting low‐k, high printing resolution, and excellent mechanical toughness hold significant promise for advanced manufacturing in semiconductor substrates and electronic packaging. Herein, a novel functional material is proposed via a one‐step UV curing process. To investigate the influence of thiol‐acrylate chain transfer behavior, a series of 3D printing inks with varying raw material ratios is formulated (Figure 1a). THEICTA features a rigid triazine ring formed by isocyanurate trimerization, which imparts significant structural stiffness and high thermal stability compared to other photocurable acrylate monomers [27]. Isobornyl acrylate (IBOA) is employed as a reactive diluent. The bulky dual ring side groups introduce substantial steric hindrance to achieve weak intermolecular interactions and large free volume [29, 30, 31]. Consequently, the introduction of IBOA endows the modified triazine‐based resin with low viscosity and low dielectric properties (Figure S1), which are crucial for high‐resolution printing and effective wave transparency. The detailed compositions are listed in Table S2.

As shown in Figure 1b, the viscosity tests indicate that all ink formulations exhibit excellent rheological behaviors (with η <150 cP), meeting the requirement of high‐resolution 3D printing. Because of the long chain structure of rubber molecules, the viscosity of the 3D printing ink slightly increases with the loading content of PSR. Before 3D printing, the monomers are thoroughly mixed and loaded into the resin vat of the 3D printer. As illustrated in Figure 1c, the trifunctional monomer THEICTA and the reactive diluent IBOA rapidly form a complex crosslinked network under 405 nm UV irradiation. The thiol‐terminated polysulfide rubber is incorporated into the network via a thiol‐acrylate chain transfer behavior. Specifically, the thiol group reacts with a propagating acrylate chain by capturing its terminal free radical. The generated thiol radical initiates the polymerization of another acrylate monomer, thereby promoting continuous chain growth. The 3D‐printed lattice sample shown in Figure 1d exhibits high printing accuracy. As observed in the SEM image, the interlayer regions of the printed structure with a wall thickness of 100 µm are tightly bonded, indicating excellent layer‐to‐layer adhesion [32]. As a supplement, tensile specimens are printed along the X, Y, and Z directions, demonstrating excellent mechanical consistency (Figure S2). The acetone swelling test results (Figure 1e, Equation S1) further confirm the degree of photopolymerization. The as‐printed structure is not fully cross‐linked, retaining only 86% of its weight after solvent exposure. After post‐curing of 15 min, the weight retention increases to 99.2%, suggesting near‐complete network formation. Compared to the mold‐cured sample with 365 nm UV treatment, the results demonstrate that ink is compatible with DLP 3D printing.

To further investigate the curing process, Raman spectroscopy is conducted. As shown in Figure 1f, characteristic peaks appear at 1640 and 2562 cm^−^^1^, which correspond to the stretching vibrations of C═C and ─SH groups, respectively. Comparing spectrum III with spectrum II, a portion of the C═C double bonds remains unreacted, while the thiol peak has disappeared, indicating that thiols are involved in chain transfer reactions. In spectrum IV, the disappearance of the C═C peak after post‐curing confirms the completion of the polymerization process.

### 2.2 Polymerization Kinetics

**FIGURE 2:**
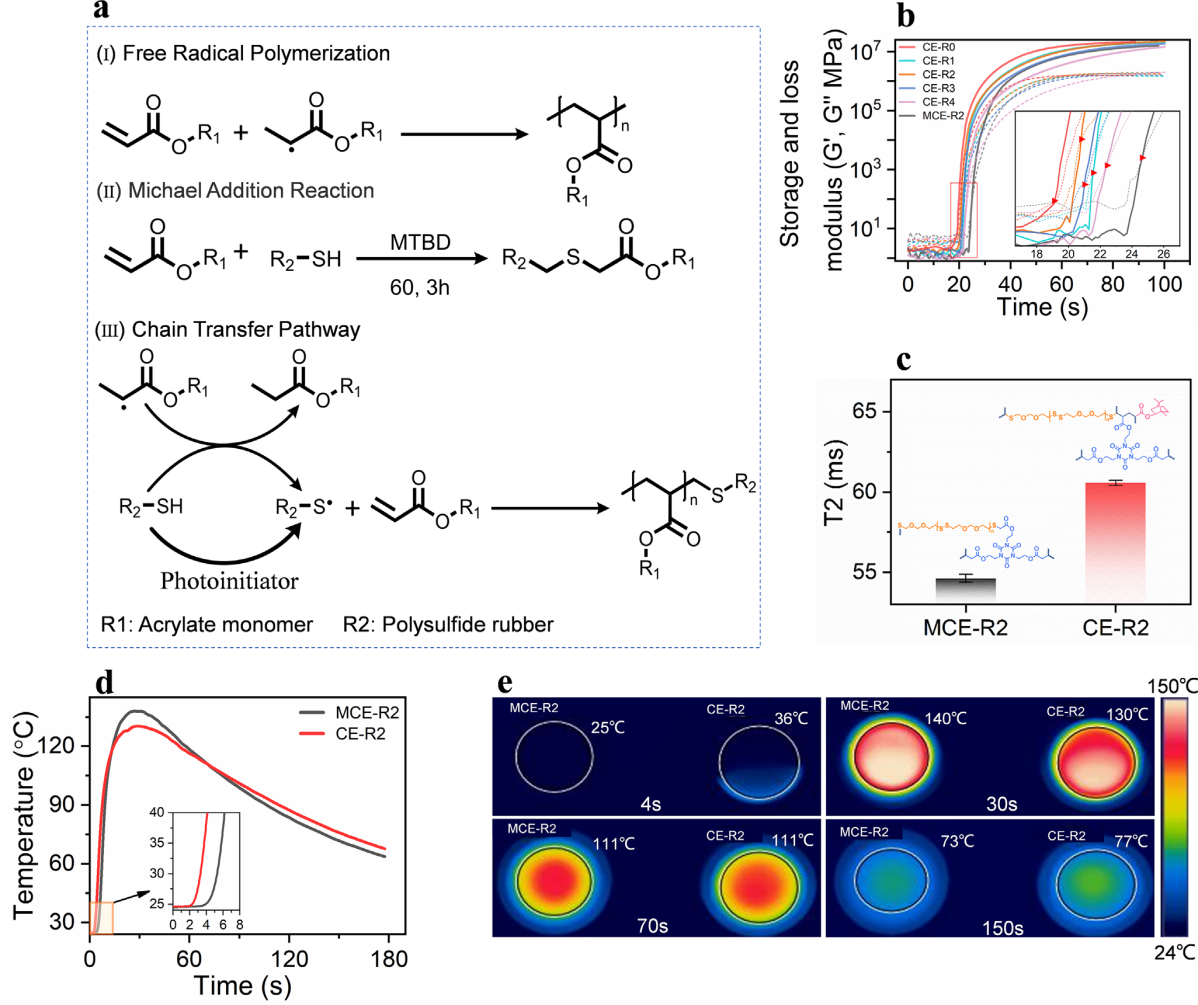
(a) Schematic illustration of the reaction mechanism and cross‐linked network obtained via free radical polymerization (I), Michael addition reaction (II), and chain transfer pathway (III). (b) The photo‐rheology of inks. (c) The transverse relaxation time of UV‐cured CE‐R2 and MCE‐R2. (d) Temperature variation of CE‐R2 and MCE‐R2 when simultaneously irradiated with UV light. (e) The local temperature is monitored by an infrared camera.

Based on the Raman spectroscopy results, it can be concluded that thiol groups are involved in chain transfer reactions and reacted with acrylate monomers and photoinitiators during the curing process. To further investigate the effect of chain transfer on the network formation and molecular aggregation behavior, a control group after the Michael addition reaction (MCE‐R2) is designed, using the base catalyst 7‐Methyl‐1,5,7‐triazabicyclo[4.4.0] dec‐5‐ene (MTBD) to initiate a nucleophilic addition reaction to consume the thiol groups in CE‐R2 group (Figure S3). As illustrated in Figure 2a, the incorporation of thiols into the free‐radical polymerization of acrylates transforms the curing mechanism into a thiol‐acrylate binary system. In this binary system involving radical polymerization and step‐growth polymerization, PSR acts as a chain‐transfer agent, enabling partial chain termination and re‐initiation [21, 33]. This topology transition intrinsically reduces crosslink density while expanding free volume.

The evolution of storage (*G′*) and loss (*G″*) moduli during UV exposure is monitored using a UV rheometer (Figure 2b). Each group is exposed to 405 nm, 20 mW/cm^2^ UV light for 10 s, and the results demonstrate that the increasing ratio of thiol to acrylate leads to a progressive delay of the gel point. Specifically, the gel points, defined as the crossover of *G′* and *G″*, are observed at 19.13, 20.82, 21.02, 21.71, and 22.38 s for networks with PSR to THEICTA weight ratios of 0, 0.3, 0.333, 0.4, and 0.5, respectively. In the case of MCE‐R2 sample, the occurrence of Michael addition reaction consumes a portion of the vinyl groups and results in a lower initial C═C density and extended gelation time [22].

Low‐field NMR is an analytical technique used to evaluate molecular mobility by monitoring the relaxation behavior of hydrogen nuclei (^1^H) in a magnetic field. Upon excitation by an external magnetic field, protons transition to higher energy states. When the magnetic field is altered, the return of these protons from the excited state to a lower energy state occurs through a process known as transverse relaxation [34, 35]. The required time of this process is referred to as the transverse relaxation time (*T_2_*). Variations of *T_2_* reflect the mobility changes of hydrogen protons associated with polymer chain segments and the crosslinking density of the material [36]. As shown in Figure 2c, the *T_2_* of MCE‐R2 and CE‐R2 are 54.5 and 61.2 ms, and the crosslinking density of MCE‐R2 and CE‐R2 are 37.3 × 10^−4^ and 30.8 × 10^−4^ mol cm^−3^, respectively. The increase of crosslinking points restricts the mobility of polymer chains by “locking” their motion, thereby leading to a shorter *T_2_*. The thiol‐mediated chain transfer process modulates the spatial conformation of polymer segments during the curing reaction, resulting in a reduction in crosslinking density. The results indicate a topology change, providing an effective pathway for in situ toughening and free volume measuring.

To evaluate the difference of polymerization process, the temperature evolution during the polymerization process is monitored using an infrared thermal camera (Figure 2d). Two models of ink, MCE‐R2 and CE‐R2, are uniformly exposed to UV light, and the infrared camera continuously records their average surface temperatures during the curing process. In the MCE‐R2 system, a portion (7.06%) of the acrylate functional groups has been consumed via the Michael addition reaction, leading to a delayed temperature rise appeared at the 3.45 s, whereas the CE‐R2 system shows a temperature increase at the 1.96 s. The rapid chain propagation of free‐radical polymerization restricts the mobility of reactive centers and reduces the termination rate constant [22], resulting in heat accumulation in the early stage of polymerization. Interestingly, although CE‐R2 contains a larger amount of concentration of C═C, the MCE‐R2 system exhibits a higher peak temperature because the thiol‐acrylate chain transfer behavior slowed the polymerization rate of CE‐R2. As shown in Figure 2e, the temperature of the CE‐R2 sample begins to rise sharply at 2 s, reaching to 36°C at 4 s, while the temperature of the MCE‐R2 sample remained nearly constant. At the 30 s, MCE‐R2 reaches the maximum temperature of 140°C, surpassing the peak value of CE‐R2 at 130°C. At the 70 s, the temperatures of both samples show an equilibrium state, after which the CE‐R2 system exhibits a slower cooling rate.

### 2.3 Dielectric Response to Thiol‐acrylate Chain Transfer Reactions

To investigate the influence of thiol‐acrylate chain transfer behavior on chain conformation and dielectric properties, broadband dielectric spectroscopy (BDS) is employed to study the molecular dynamics of the cured network by distinguishing the relaxation process. It is well established that dielectric relaxation is mainly classified into two regions, above and below the glass transition temperature (*T_g_*), where the chain segment motion modes differ distinctly. Above *T_g_*, the polymer chains exhibit active segmental motions with dielectric relaxation predominantly governed by α‐relaxation. In contrast, the motion of the main chains is frozen below the *T_g_*, and the dielectric relaxation is dominated by side chain β‐relaxation [37].

**FIGURE 3:**
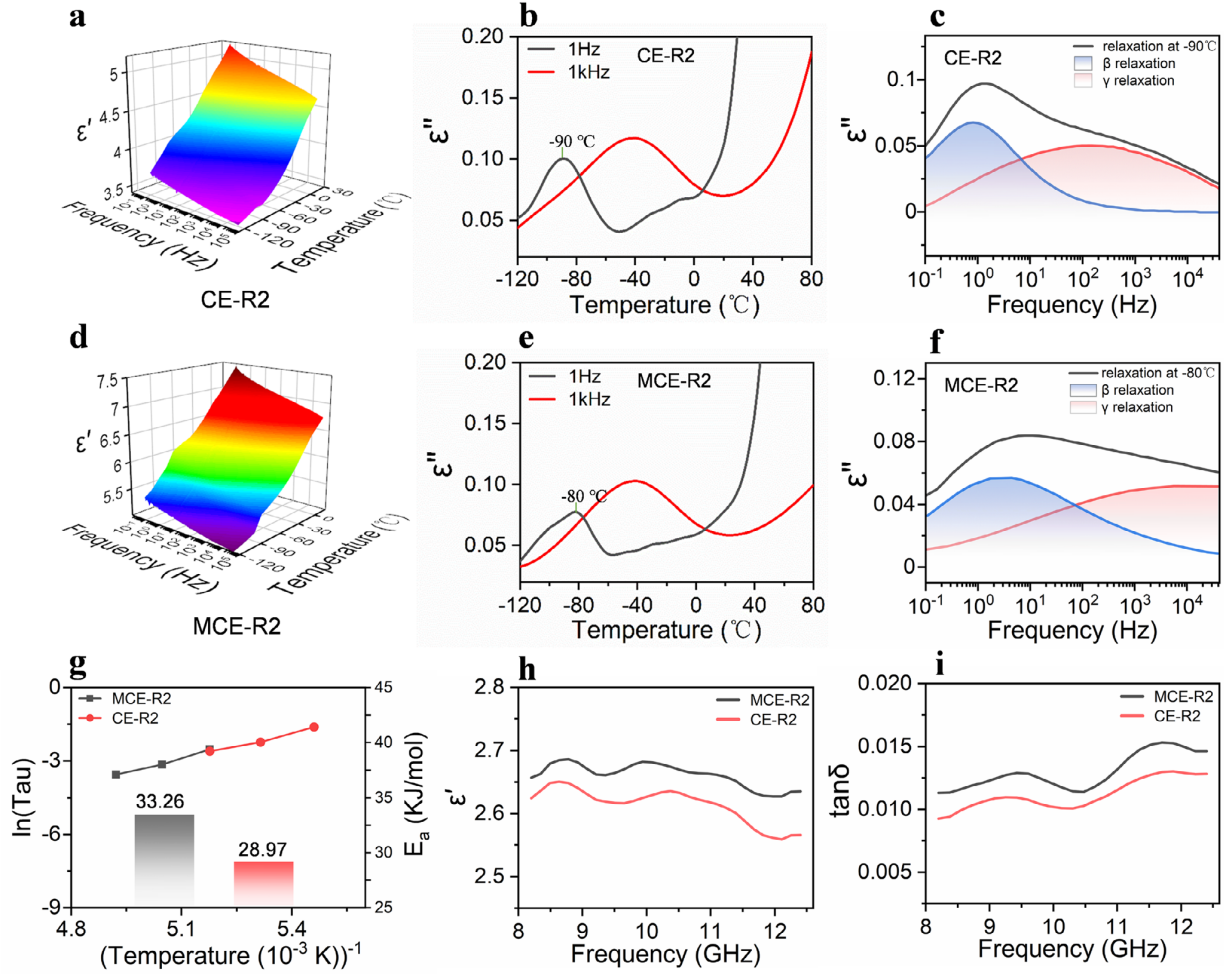
(a) Dielectric constant as a function of frequency and temperature for CE‐R2. (b) Dielectric loss as a function of temperature at 1 kHz and 1 Hz for CE‐R2. (c) Dielectric loss spectra of CE‐R2 fitted using a combination of two H‐N equations at −90°C. (d) The *ε′* of MCE‐R2. (e) The *ε″* of MCE‐R2. (f) The dielectric loss spectra of MCE‐R2 at −80°C. (g) Average relaxation time (*Tau*) and activation energies (*E_a_*) of β‐relaxation as a function of temperature for CE‐R2 and MCE‐R2. (h,i) *ε′* and tanδ of CE‐R2 and MCE‐R2 in the X band, respectively.

Accordingly, a series of frequency sweeps at different temperatures is conducted for the cured samples of MCE‐R2 and CE‐R2. As shown in Figure 3a–d, dipole reorientation is largely frozen at low temperatures, with the temperature increase from −120°C to 20°C, the enhancement of dipole orientation polarization leads to an increase in the real part of the dielectric constant. The dielectric relaxation loss peak of CE‐R2 and MCE‐R2 also shifts toward higher frequencies as the temperature rises (Figure S4). Moreover, the samples exhibit frequency dependence [38]. As the frequency increases, the polarization response lags behind the rapid alternating of the electric field, resulting in a decrease of *ε′*. It is noteworthy that MCE‐R2 consistently exhibits a higher ε′ than that of CE‐R2, indicating that the chain transfer behavior reduces the dipole density and increases the free volume of the chain segment. Figure 3b–e presents the variation of the imaginary part of the dielectric constant for samples at 1 Hz and 1 kHz. At low temperatures, the motion of the polymer backbone segments is frozen, and the secondary relaxation peaks are observed at both frequencies. The compared performance reveals a clear difference of the relaxation peaks at 1 Hz, with CE‐R2 and MCE‐R2 exhibiting peak maxima at −90°C and −80°C, respectively.

To quantitatively analyze the dielectric relaxation processes, the frequency dispersion curves of dielectric constant are fitted by two Havriliak–Negami models to resolve the relaxation type of polymer chains (Equations S3 and S4). As shown in Figure 3c–f, the β‐relaxation process (local motion of side chains) and the γ‐relaxation process (vibrations of ester groups and thioether moieties) [39]. are extracted from the H‐N equations and plotted as functions of temperature. It can be observed that γ‐relaxation is faster than β‐relaxation. The relaxation times (*Tau*) and activation energies (*E_a_*) are further analyzed using the Arrhenius equation (Equations S5 and S6). As presented in Figure 3g, the β‐relaxation process of the MCE‐R2 sample exhibits both a higher activation energy (*E_a_*) and a higher relaxation peak temperature compared with CE‐R2, confirming that thiol‐acrylate chain transfer behavior increases the free volume between chain segments and reduces intermolecular interactions. In the gigahertz (GHz) frequency range, dipole polarization lags behind the variations of the electromagnetic field, and electronic polarization becomes dominant. Figure 3h,i displays the dielectric constant and dielectric loss of MCE‐R2 and CE‐R2 in the X‐band (8–12.4 GHz). For MCE‐R2 group, the absence of chain transfer behavior during photocuring leads to higher cross‐linking density and tight the stacking of triazine rings. This would enhance the delocalization of π‐electron, thereby resulting in an increase in *ε′* and a shift of the dielectric loss peak toward higher frequencies. In contrast, the CE‐R2 sample possesses larger free volume, as the polysulfide segments expand the spacing between triazine rings, thereby reducing electron density. As a result, both *ε′* and tanδ of CE‐R2 are lower.

### 2.4 Mechanical Enhancement

**FIGURE 4:**
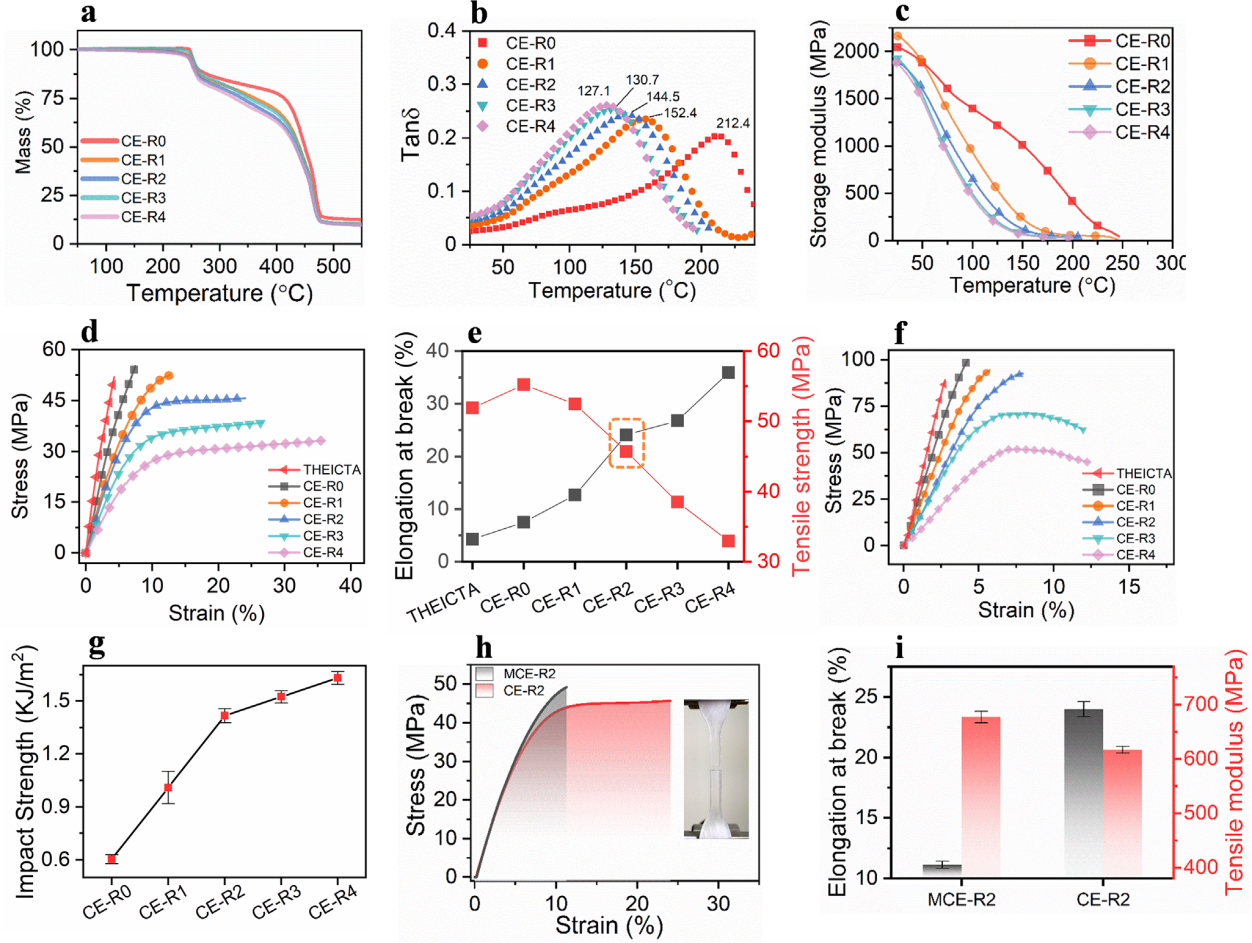
(a) The Thermogravimetric Analysis of the CE‐R resins. (b) The loss tangent (tan δ) of dynamic mechanical analysis. (c) The storage modulus. (d) The tensile test curves. (e) Comparison of elongation at break and tensile strength. (f) The flexural test curves. (g) The impact strength of resins. (h) Comparison of toughness for CE‐R2 and MCE‐R2. (i) Comparison of elongation at break and tensile modulus for CE‐R2 and MCE‐R2.

To analyze and optimize the PSR content, a series of inks is formulated and 3D printed (Table S2). As shown in Figure 4a, the thermal stability of the cured resins is assessed by thermogravimetric analysis (TGA). The first stage of weight loss at 245.77°C is attributed to the degradation of ester side groups. The second stage at 263.23°C corresponds to the thermal decomposition of the polysulfide rubber. As the rubber content increases from CE‐R0 to CE‐R4, more disulfide and thioether bonds are introduced into the system, causing accelerated mass loss in the corresponding temperature ranges. In the third stage, beginning around 400°C, a sharp mass loss caused by the breakdown of the polymer backbones is observed.

To further evaluate the thermomechanical performance, a DMA test is conducted to determine the glass transition temperature (*T_g_*) and storage modulus of the CE‐R resins. As shown in Figure 4b, owing to the flexible long‐chain structure of the rubber, the *T_g_* decreases progressively with increasing PSR content. For the neat group without rubber substance, CE‐R0 exhibits the highest *T_g_* at 212.4°C. In contrast, as the rubber increased from CE‐R1 to CE‐R4, the *T_g_* values gradually decrease from 152.4°C to 127.1°C. As temperature increases, chain segment mobility becomes more pronounced to induce a reduction in storage modulus (Figure 4c).

Due to the conformational changes caused by chain‐transfer behavior, the mechanical properties are largely improved. As shown in Figure 4d–f, both the elongation at break and the flexural deflection at break exhibit an increasing trend with higher PSR content, indicating an enhancement of the toughness. However, when the PSR content reaches 40 wt.% relative to THEICTA (CE‐R3), both the tensile strength and flexural strength begin to decline. Notably, as summarized in Figure 4e, when the mass ratio of THEICTA to PSR is 3:1 (CE‐R2), the mechanical performance reaches an optimal balance with the elongation at break and tensile strength achieve 24% and 45.68 MPa, respectively. Compared with the neat THEICTA and CE‐R0 groups, the tensile toughness increased from 1.2 and 2.26 to 8.87 MJ/m^3^, corresponding to improvements of 640.5% and 286%, respectively. (Figure S5a). The Poisson's ratio of CE‐R2 is 0.4 (Figure S6, Equation S7), calculated by 2D Digital Image Correlation (2D‐DIC) system (Video S1). After exceeding the yield strength, a distinct “necking” stage demonstrates a significant enhancement in toughness. As shown in Figure 4g, the Charpy impact testing reveals that the impact strength increases from 0.6 to 1.63 kJ m^−2^ with increasing rubber content, indicating that the addition of the PSR effectively suppresses crack propagation under impact loading. On the one hand, this remarkable improvement in toughness is attributed to the excellent polar compatibility between the PSR and the triazine resin, which leads to the formation of an interpenetrating network between the flexible rubber chains and the rigid triazine resin chains after curing. On the other hand, the terminal thiol groups of the PSR form covalent bonds with the triazine resin through chain transfer reactions, which further promotes compatibility and energy dissipation.

As the thiol groups in the MCE‐R2 system are consumed via Michael addition reactions, no chain‐transfer behavior occurs during the DLP printing. The toughness enhancement in MCE‐R2 is only attributed to the incorporation of flexible rubber segments, whereas in CE‐R2, the toughness enhancement is derived from flexible chains and the in situ thiol‐acrylate chain transfer behavior synergistically. A comparison reveals that the toughness of CE‐R2 is notably superior to that of MCE‐R2 in tensile testing (Figure 4h). Although MCE‐R2 exhibits a slightly higher tensile modulus (677 MPa) than CE‐R2 (617 MPa), its elongation at break is only 11.13% (Figure 4i). Based on the integral area under the stress–strain curves, the tensile toughness of CE‐R2 is enhanced by 162% compared to MCE‐R2 (3.42 MJ m^−3^) (Figure S5b). This suggests that the toughening effect mainly arises from optimized crosslink density under identical compositions.

**FIGURE 5:**
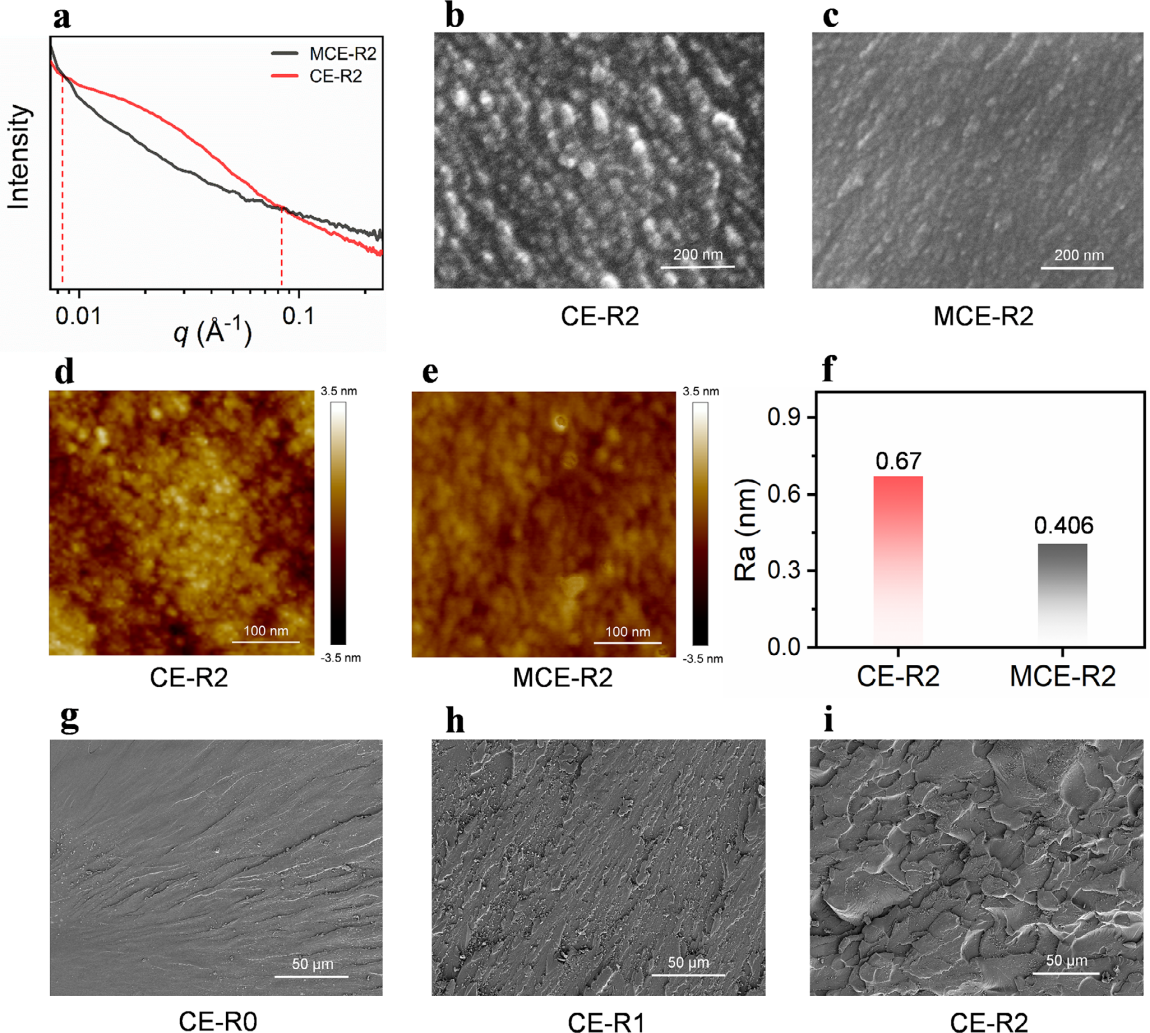
(a) 1D SAXS scattering (1D SAXS) profile of CE‐R2 and MCE‐R2. (b,c) The SEM images of CE‐R2 and MCE‐R2 fragmented by liquid nitrogen. (d,e) Atomic force microscopy surface topographies of CE‐R2 and MCE‐R2. (f) Comparison of arithmetic average roughness. (g–i) Impact section morphology of CE‐R0, CE‐R1 and CE‐R2, respectively.

To verify the in situ toughening mechanism of thiol‐acrylate chain transfer from a microscopic perspective, small‐angle X‐ray scattering (SAXS) analysis is performed on CE‐R2 and MCE‐R2 resins. As shown in the 1D SAXS profile (Figure 5a), a broad scattering peak is observed in the *q* range of 0.0085–0.085 Å^−1^ for the CE‐R2 sample. According to Bragg's law [40], the corresponding microstructures sizes range from approximately 7.3 to 74 nm, which attributes to the change of topological structure induced by the chain transfer behavior during DLP printing.

SEM images (Figure 5b,c) further reveal significant differences in aggregate structures of the chain segment between the two ink systems. For CE‐R2, the thiol‐acrylate chain transfer reaction reduces the crosslinking density and alters the segmental topology, leading to a distinct “sea‐island” morphology [41]. In contrast, for the free‐radical polymerization ink system of MCE‐R2, the aggregate structures demonstrate a more homogenous morphology. These results suggest that the chain transfer reaction enables controlled formation of aggregated microdomains, providing an effective in situ toughening pathway under mechanical deformation.

Figure 5d,e presents AFM images of the samples, further confirming that CE‐R2 exhibits an aggregated, clustered morphology. In contrast, the corresponding MCE‐R2 is characterized by the absence of effective mechanically dissipative microstructural features. The arithmetic average roughness (Ra) also reveals that MCE‐R2 possesses a notably smoother surface topography (Figure 5f). Finally, as shown in Figure 5g,h,i, the impact fracture surfaces of samples with different toughening agent contents are characterized by SEM. The CE‐R0 group exhibits a relatively smooth fracture surface, indicating typically brittle fracture behavior. With the gradual addition of PSR, the fracture morphology of CE‐R1 group transitions from a smooth “river‐like” pattern to a “feather‐like” pattern, representing the brittle‐to‐ductile transition point [42]. Further, rough cracks and distinct dimple structures are observed in the CE‐R2 group, which are key factors for enhanced toughness [43].

### 2.5 Simulation and Structural Optimization

**FIGURE 6:**
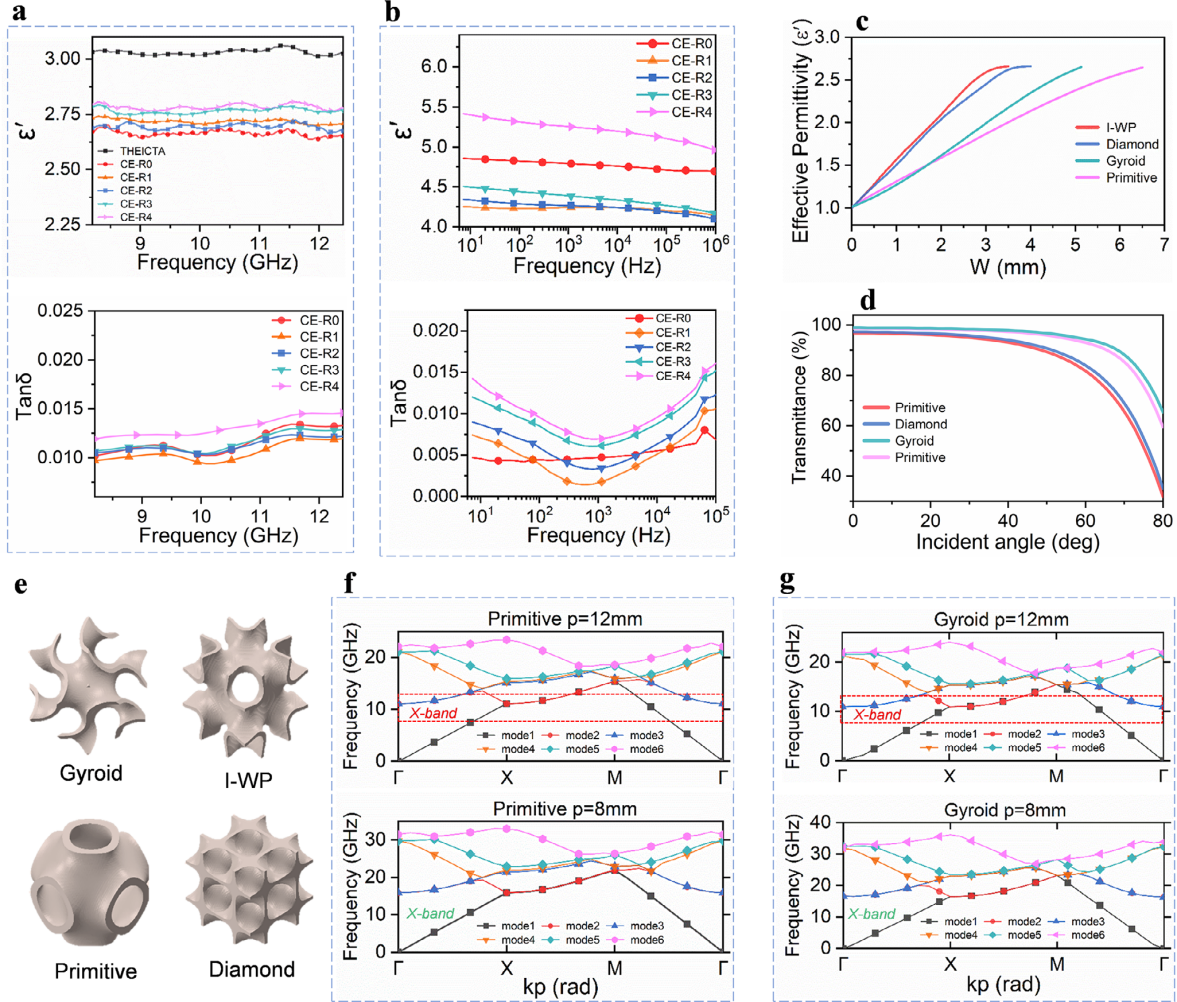
(a) The *ε′* in the X‐band and low‐frequency region of CE‐R resins. (b) The *tanδ* in the X‐band and low‐frequency region of CE‐R resins. (c) The effective permittivity variation according to the change of lattice wall thickness (parameter *W*). (d) The transmittance according to the change in incident angle. (e) The unit cell of TPMS structures. (f,g) Dispersion diagram of Primitive and Gyroid structures.

To achieve impedance matching and efficient signal transmission, low‐*k* and low *tan δ* of the matrix material are particularly important for the wave transparency structure. According to the Debye equation [44, 45], dielectric properties are governed by dipole density, polarizability, and dipole moment. In the X‐band, the incorporation of IBOA molecules introduces large free volume, leading to a sharp decrease in *ε′* from 3.03 for THEICTA to 2.61 for CE‐R0. As the PSR content extends from CE‐R1 to CE‐R4, the *ε′* shows a slight increase, because the thioether bonds in the PSR segments possess relatively high polarity (Figure 6a). In all of these experimental groups, CE‐R2 demonstrates remarkable mechanical properties and optimal dielectric properties, along with *ε′* of 2.66 and *tan δ* of 0.0103 at 10 GHz. Moreover, with the increase in the PSR content, the dielectric loss shows a trend of first decreasing and then increasing (Figure 6b). This phenomenon can be explained by the fact that when the mass ratio (PSR:THEICTA) reaches 1:2, the influence of the polar chain segments becomes dominant over that of the free volume. A similar trend is observed in the low‐frequency region. The incorporation of PSR reduces the dipole density, causing the dielectric constant to initially decrease to 4.25 of CE‐R2 and then increase gradually. Meanwhile, the dielectric loss remains below 0.015 across all samples, with CE‐R2 remaining below 0.01.

Based on the aforementioned ink with chain transfer behavior, which has superior printability and dielectric properties, it can be used for the preparation of complex curved transparent structures by DLP printing. Triply periodic minimal surface (TPMS) is a novel class of porous architectures (Table S3). Owing to the unique high‐porosity open‐cell geometry, TPMS structures also exhibit significant potential in electromagnetic wave‐transmitting structures. In this study, four kinds of TPMS lattice types (Gyroid, Primitive, I‐WP, and Diamond) are selected for designing the curved structures with outstanding dielectric and mechanical performances (Figure S7). Based on the electromagnetic parameters of the CE‐R2 sample, equivalent dielectric constant calculations and angular transmittance simulations are performed by Floquet theory in CST Studio Suite. The effective dielectric constants at 10 GHz are obtained using the S‐parameter inversion method (Equation S11) for structure with different wall thicknesses *W* (mm). As shown in Figure 6c, the equivalent dielectric constant of all lattice types increases with wall thickness, and gradually approaches to the intrinsic dielectric constant of the material. Among these classical structures, the Primitive and Gyroid lattices exhibit superior dielectric performance due to their higher porosity. The electromagnetic transmittance under TE polarization is calculated from parameter *S_21_* with the *W* of 0.5 mm (Equation S12). As depicted in Figure 6d, these two lattices also demonstrate higher transmittance at different incidence angles. Notably, the Gyroid lattice maintains 94.1% transmittance at 60° incidence angle and 65.4% at 80°. Figure 6e presents the unit cells of four kinds of TPMS lattices.

In order to optimize the lattice constant, the dispersion diagram is employed to illustrate the relationship between the propagation constant and frequency. The diagram is critical for accurately describing the phase velocity and group velocity characteristics of periodic structures, and aids in identifying the allowed modes for a given operating frequency band. To construct the dispersion diagram, an eigenmode analysis is performed in Equation S13. As shown in Figure 6f,g, the dispersion diagrams of Gyroid and Primitive lattice reveal that the TE0 (mode 1) and TM0 (mode 2) modes dominate across two structures within the frequency range of 0–25 GHz. Specifically, when the lattice period *p* = 12, higher‐order modes are observed at the Γ point in both Primitive and Gyroid lattices. These higher‐order modes in the target X‐band, commonly known as diffraction gratings, will lead to the scattering of electromagnetic waves and deteriorate the overall transmission performance [46]. To mitigate this issue, the operational frequency range can be extended by reducing the lattice period. When the period *p* = 8 mm, the higher‐order modes shift to 15.9 and 17.2 GHz for the Primitive and Gyroid lattices, which are outside the target frequency band.

### 2.6 Applications of the Ink

**FIGURE 7:**
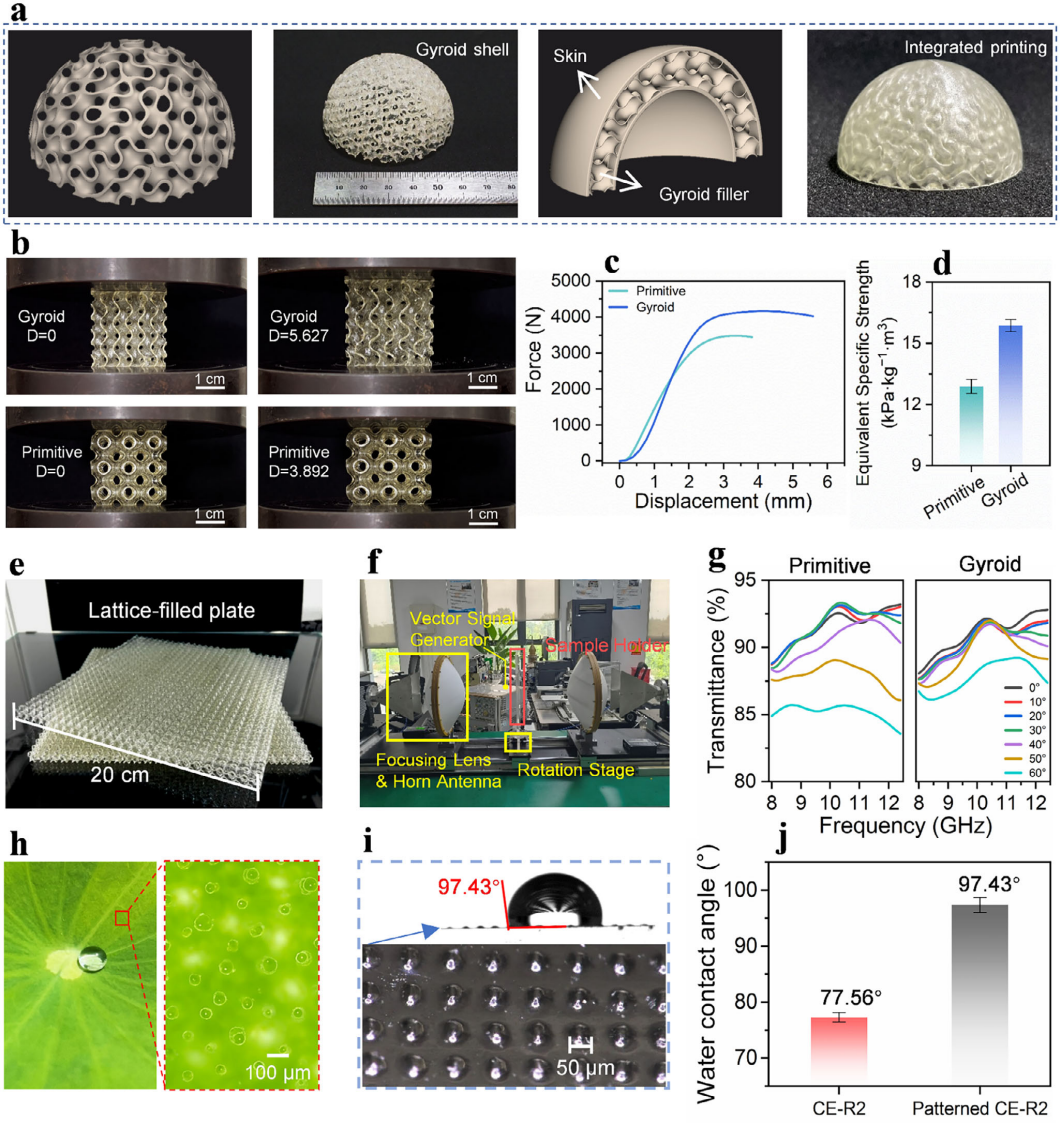
(a) Modeling and 3D printed demonstration of electromagnetic wave‐transmitting structures. (b) The compression tests of 3 cm × 3 cm × 3 cm Gyroid and Primitive lattice. (c) The force‐displacement curves. (d) The equivalent specific strength of printed Gyroid and Primitive lattices. (e) 3D printed 20 cm × 20 cm lattice‐filled plates. (f) The Vector Network Analyzer (VNA) and free‐space measurement system. (g) The transmittance contrast of Gyroid and Primitive lattice‐filled plate. (h) The micro structure of lotus leaves. (i) 3D printed surface array structure. (j) The improvement of water contact angle.

In the practical design and fabrication of wave‐transmitting radomes, impedance mismatch caused by the different materials of the skin and the filler remains a critical challenge. By utilizing the optimized CE‐R2 ink formulation, an integrated radome structure is fabricated (Figure 7a). The radome is comprised of skin (1 mm thick) and Gyroid lattice filler (8 mm period). The integrated printing of the radome demonstrates the capability of the ink to achieve precise 3D printing. Subsequently, as shown in Figure 7b, to compare the mechanical load‐bearing performance, two 3 cm× 3 cm samples are printed for compression testing (Figure S8, Video S2). As illustrated in Figure 7c, the Primitive lattice exhibits a higher initial compressive modulus, whereas the Gyroid lattice demonstrates greater compressive strength and superior energy absorption capacity. Besides, the equivalent specific strengths of the Primitive and Gyroid lattice structures are 12.88 and 15.86 kPa kg^−1^m3, respectively (Figure 7d, Equation S14). Under the external force compression, the Gyroid TPMS structure shows a relatively uniform stress distribution, while the Primitive lattice experiences pronounced stress concentration at the curved pore walls [47, 48].

In the following, two types of lattice‐filled plates measuring 20 cm× 20 cm are printed (Figure 7e), and their actual transmission performance are tested using a free‐space measurement system. As depicted in Figure 7f, a pair of horn antennas and a focusing lens are mounted on a rail system. The antennas are connected to a vector network analyzer (VNA). According to the test results (Figure 7g), under normal incidence, the vertically aligned through‐holes of the Primitive lattice structure contribute to achieve a high transmittance of 92.5% at 10 GHz. Due to the diagonal through‐holes characteristic, the Gyroid lattice maintains a high transmittance of approximately 92% at 10 GHz for incident angles up to 50°, making it more suitable as a filler structure in wide‐angle scanning radomes.

Furthermore, in practical environments, water absorption usually affects the dielectric performance of low‐*k* materials. Thus, a hydrophobic microstructure array inspired by lotus leaves is printed on the outer surface of the radome (Figure 7h,i). Moreover, the thioether and disulfide bonds in PSR can also decrease the surface energy to enhance the hydrophobicity (Figure S9). As shown in Figure 7j, the surface patterning increases the water contact angle of CE‐R2 resin from 77.56° to 97.43°, thereby achieving a transition from hydrophilicity to hydrophobicity.

## 3 Conclusions

In summary, integrating PSR into modified triazine resins can markedly in situ enhance the toughness, dielectric properties and printability. The dielectric constant of CE‐R2 drops from 3.03 to 2.66 and remains low dielectric loss across a wide frequency range. The PSR in situ toughening resins also exhibit notable mechanical improvements, with the optimal elongation at break increasing by 462.4% and toughness by 640.5% compared to the pure THEICTA. The effects of chain transfer behavior offer a promising strategy for designing low‐*k* electronic materials. Furthermore, the successful application in 3D printing demonstrates the potential for high precision fabrication of wave‐transparency curved structures. Nevertheless, the thermotolerance remains a challenge for CE‐R resins under extreme conditions, underscoring the imperative for further research.

## Conflicts of Interest

The authors declare no conflict of interest

## Supporting information




**Supporting File 1**: advs74759‐sup‐0001‐SuppMat.docx.

**Supporting File 2**: advs74759‐sup‐0002‐VideoS1.mp4.

**Supporting File 3**: advs74759‐sup‐0003‐VideoS2.mp4.

## Data Availability

The data that support the findings of this study are available from the corresponding author upon reasonable request.
