# Supplementary material for: Precise Tailoring 3D Printed In Situ Toughening Low‐k Microwave Transparent Structure via Thiol‐acrylate Chain Transfer Behavior
Source: Adv Sci (Weinh). 2026 Mar 10;13(29):e74759. doi: 10.1002/advs.74759 (PMC13205786; doi:10.1002/advs.74759)
Supplement: Supplementary file 1 — Supporting File 1: advs74759‐sup‐0001‐SuppMat.docx. [file ADVS-13-e74759-s003.docx]

Supporting Information

**Precise Tailoring 3D Printed *In-situ* Toughening Low-*k* Microwave Transparent Structure *via* Thiol-acrylate Chain Transfer Behavior**

*Ce Bian^[a,b]^, Kai Zheng^[a,b]^, Ruoyu Chen^[a,b]^, Wansong Gu^[a,b]^, Guanxiang Zhong^[a,b]^, Difei Liang^[a,b]^* Longjiang Deng^[a,b]^, Hetao Chu^[a,b]^**

[a] National Engineering Research Center of Electromagnetic Radiation Control Materials, University of Electronic Science and Technology of China (UESTC)

Chengdu 611731, China

[b] Key Laboratory of Multispectral Absorbing Materials and Structures of Ministry of Education, University of Electronic Science and Technology of China (UESTC)

Chengdu 611731, China
E-mail: [dfliang@uestc.edu.cn](mailto:dfliang@uestc.edu.cn) (D. Liang); [chuht@uestc.edu.cn](mailto:chuht@uestc.edu.cn) (H. Chu)

# Experimental Section

## Materials

Tris(2*-*hydroxyethyl)-isocyanurate triacrylate (THEICTA) and iso*-*Bornyl acrylate (IBOA) were purchased from Shanghai Adamas Reagent Co., Ltd. The photoinitiator Phenylbis(2,4,6*-*trimethylbenzoyl)-phosphine oxide (Irgacure 819) and catalyst 7*-*Methyl-1,5,7*-*triazabicyclo[4.4.0]dec-5*-*ene (MTBD) were purchased from Shanghai Aladdin Bio - Science & Technology Co., Ltd. The liquid polysulfide rubber (JLY-121, molecular weight ~1,000 with thiol groups terminated) was obtained from Sinochem Energy Co., Ltd. All chemical reagents were used as received unless otherwise noted.

## Preparation of Ink CE-R0 to CE-R4

The monomers and catalyst were stepwise mixed to obtain the 3D printing ink. First, 100 g THEICTA was added into a 250 mL brown beaker, then the beaker was heated to 80°C to melt the waxy solid. Next, 49.2 g diluent IBOA (the molar ratio of THEICTA and IBOA is 1:1), which also functions as a co-monomer, was tipped into the beaker as well. According to Table 1, different qualities of liquid polysulfide rubber were slowly added into acrylic ester monomer while vigorously stirring. After 20 min of rapid stirring, the mixed monomers were cooled to room temperature, and 0.736 g photoinitiator I-819 (0.5 wt% of the acrylic monomers) was enrolled to gain a printable ink. The beaker was finally evacuated in a vacuum oven for 30 min to remove internal oxygen.

## Table S1. The comparison of dielectric properties of representative reported photopolymer systems.

| Composition | Frequency | ε′ | tan δ | Ref. |
| --- | --- | --- | --- | --- |
| Commercial acrylate photopolymer | 10 GHz | 3.15 | 0.02 | - |
| Interpenetrating network CE resin | 8-12 GHz | 2.8-3.2 | 0.002-0.005 | ^[1]^ |
| UV curable naphthalene resin | 0.1 GHz | 2.8 | 0.009 | ^[2]^ |
| Fluorinated methacrylate photopolymer | 10 GHz | 2.75 | 0.01 | ^[3]^ |
| Epoxy-acrylate-based photopolymer | 8 GHz | 2.7 | 0.005 | ^[4]^ |
| This work | 10 GHz | 2.66 | 0.008 | - |

## Table S2. The composition of CE-R Resins (g)

|  | THEICTA | IBOA | PSR | I-819 |
| --- | --- | --- | --- | --- |
| CE-R0 | 10 | 4.92 | - | 0.0746 |
| CE-R1 | 10 | 4.92 | 3 | 0.0746 |
| CE-R2 | 10 | 4.92 | 3.33 | 0.0746 |
| CE-R3 | 10 | 4.92 | 4 | 0.0746 |
| CE-R4 | 10 | 4.92 | 5 | 0.0746 |

## Preparation of Pre-reaction Ink MCE-R2

After sufficiently blended the monomers mixture (THEICTA, IBOA, and liquid polysulfide rubber) above, MTBD, which accounts for 0.02% of the total mass was dissolved in 0.5 mL of acetone and then added dropwise to the mixed resin while stirring. The mixture was stirred at a temperature of 45 ℃ for 2 h. Then, trace amounts of solvents and catalysts were evaporated at the temperature of 75 ℃. Finally, the photoinitiator I-819 was enrolled and the pre-reaction ink was evacuated for 30 min.

## Three-dimensional Printing and Post-curing

3D printing was performed with a DLP 3D printer (SprintRay-Pro) with the wavelength of 405 nm. The layer thickness was set to be 50 μm, and the UV light intensity and exposure time were adjusted based on the polymerization of printing ink. After the 3D printing, half-cured resin was detached from the working platform and deposited into a UV light oven. A well crosslinked transparent solid object was obtained after post-curing procedure for 10 min.

## Characterization

### Material Characterization

A confocal microscopic Raman spectrometer (Renishaw inVia, UK) with 633 nm laser was used to verify the reaction degree of functional groups at different states. The viscosities of samples were tested by a viscometer (Brookfield DV-II Pro, America). The weight retention rate was determined by swelling experiments with acetone for 72 h. The products were then dried in a vacuum oven at 80 ℃ for 24 h. An optical rheometer (MCR302, Anton Paar) was used to perform the rheological behavior under UV irradiation, including the storage modulus, loss modulus, and gelation time. The transverse relaxation times (*T_2_*) of cured resins were measured using a variable-temperature, low-field nuclear magnetic resonance (NMR) analysis (VTMR20-010V-I, Suzhou Niumag Corporation, China). The Carr-Purcell-Meiboom-Gill sequence was employed for data acquisition. The temperature variation of resins during UV-curing process was monitored by an infrared thermal camera (FLIR T640, America). Scanning electron microscopy (SEM) images of cross-sectional morphology were obtained with a field emission microscope (ZEISS Sigma 300, Gekrmany). All samples were coated with platinum by sputtering prior to observation. The nanoscale structure of the cured sample was analyzed by Small Angle X-ray Scattering instrument (SAXSpoint500, Anton Paar). Water contact angle was measured on a dynamic contact angle measuring instrument (JC2000D1, POWEREACH, China).

### Mechanical Characterization

The tensile strength and three-point bending tests of resins cured with different components were measured using a universal testing machine (HZ-1004B, Dongguan Lixian Instrument Technology Co., Ltd., China). At least three specimens were tested for each sample to calculate average values. The DIC test was involved to calculate the Poisson's ratio simultaneously with the tensile test, during which the surface was photographed using a high-speed camera (Shimadzu HPV-X2, Japan). Images were captured 150 ms intervals before sample's failure. The VIC-2D system software (Correlated Solutions, USA) was used to calculate the local strain relative to a specified region of interest (ROI). The Charpy pendulum impact test was performed on a pendulum testing machine (CEAST 9050, INSTRON, Italy) using notched specimens with a V-notch angle of 45° and a notch depth of 2 mm derived from ASTM D256 standards.

### Dielectric Characterization

The complex permittivity of UV-cured resins were measured over a frequency range from 10 Hz to 10^5 Hz using a Concept 50 Broadband Dielectric Spectroscopy system (Novocontrol Technologies, Germany). The complex permittivity in X-band (8.2 GHz to 12.4 GHz) was measured using the rectangular waveguide method with an vector network analyzer (Agilent PNA N5230A, America). The angle scanning electromagnetic transmittance of the printed lattice-filled plate was completed by connecting a vector network analyzer to a free-space measurement system.

### Thermal Characterization

Thermogravimetric (TG) analysis was conducted using a simultaneous thermal analyzer (Netzsch STA449F3, Germany). The samples were heated from 25 °C to 550 °C under a nitrogen atmosphere at a heating rate of 10 °C/min. Dynamic mechanical analysis (DMA) was carried out by an apparatus (DMA Q850, TA, America) with scans conducted from 25 °C to 260 °C under N_2_ atmosphere at a heating rate of 5 °C/min and a frequency of 1 Hz. The test adopted a single cantilever mode with an amplitude of 5 μm and the sample dimensions were 40 mm×10 mm×3 mm.

# Equations and Analysis

## Weight Retention.

Weight retention rate (*R*) illustrates the curing rate of 3D printed sample, which is calculated according to the following equation:^[5]^

$$\begin{aligned} \text{R }\text{= }\frac{\text{w}}{\text{w}_{\text{0}}}\text{ × 100\%}\#\text{(1)} \end{aligned}$$

where $\text{w}_{\text{0}}$ is the initial mass of samples, and $\text{w}$ refers to the mass after 72 hours of swelling in acetone.

## Analyses of the Dielectric Spectra.

The dielectric spectra were analyzed using the Havriliak–Negami (H-N) model,^[6-7]^ which describes the frequency dependence of the complex dielectric permittivity (*ε**) via the following equation:^[8]^

$$\begin{aligned} \text{ε}^{\text{*}}\left( \text{ω} \right)\text{ }\text{=}\text{ }\text{ε}_{\text{∞}}\text{ +}\text{ }\frac{\text{∆}\text{ε}}{\left[ \text{1 +}\text{ }\left( \text{iω}\text{τ}_{\text{HN}} \right)^{\text{α}} \right]^{\text{β}}}\#\text{(2)} \end{aligned}$$

Here, *Δε* = *ε_s_−ε_∞_* represents the dielectric strength, where *ε_s_* and *ε*_∞_ are the static (fully relaxed) and high-frequency (unrelaxed) dielectric constants, respectively. And τ_HN_ denotes the characteristic relaxation time. The parameters *α* and *β* (0 < *α*, *αβ* ≤ 1) account for the symmetric and asymmetric broadening of the dielectric loss peak. The relation between *τ_HN_* and average relaxation time *τ_max_* is given by the following equation:^[9]^

$$\begin{aligned} \tau_{\max}=\tau_{\mathrm{HN}} \left[ \sin\frac{\pi\alpha\beta}{2\left( 1+\beta\right)} \right]^{\frac{1}{\alpha}}\left[ \sin\frac{\pi\alpha}{2\left( 1+\beta\right)} \right]^{-\frac{1}{\alpha}}\#\text{(3)} \end{aligned}$$

$$\begin{aligned} f_{\max}=\frac{1}{2\pi\tau_{\max}}\#\text{(4)} \end{aligned}$$

where *f*_max_ is the frequency at which *ε″* passes through the maximum value. The relation between the average relaxation time *τ*_max_ and the temperature is Arrhenius-like and can be described by

$$\begin{aligned} \text{τ}_{\text{max}}\text{ }\text{=}{\text{ }\text{τ}}_{\text{0}}\text{ }\text{exp}\left( \frac{\text{E}_{\text{α}}}{\text{RT}} \right)\#\text{(5)} \end{aligned}$$

where *E*_a_ is the activation energy and *τ_0_* is a proportionality constant. We refer to the value obtained from the maximum in the dielectric loss modulus as the average relaxation time *τ*_max_. This characteristic relaxation time can be correlated with the temperature through the Vogel-Fulcher-Tamman (VFT) equation:^[10]^

$$\begin{aligned} \tau_{\max}=\tau_{0} \exp\left( \frac{B}{T-T_{0}} \right)\#\text{(6)} \end{aligned}$$

where *τ*_0_ and *B* are empirical parameters and *T*_0_ is the so-called Vogel temperature.

## Poisson’s Ratio

The local strain in the tensile specimen was captured by a DIC camera and analyzed using VIC-2D software (Video 1). The sample surface was prepared with randomly distributed speckles of approximately 1 mm in size, and images were captured by high-speed camera at a rate of 300 frames per second. The Poisson’s ratio (*ν*) was calculated according to the following equation:

$$\begin{aligned} \text{ν }\text{= -}\frac{\text{εxx}}{\text{εyy}}\#\text{(}\text{7}\text{)} \end{aligned}$$

Here, $\text{εxx}$ denotes the transverse strain (the strain perpendicular to the loading direction), and $\text{εyy}$ represents the axial strain (the strain along the loading direction).

## Design of Triply Periodic Minimal Surface (TPMS) Structures

The 3D-printed wave-transmitting structures were designed through implicit equations. The implicit equations of four commonly used types of TPMS are shown below:^[11]^

**Table S3.** Mathematical expression of TPMS structures

| TPMS | Mathematical expressions |
| --- | --- |
| Gyroid type | $\text{ϕG(}\text{x}\text{, }\text{y}\text{,}\text{z}\text{) = sin(}\text{λx}\text{) cos(}\text{λy}\text{)+sin(}\text{λz}\text{) cos(}\text{λx}\text{)+sin(}\text{λy}\text{) cos(}\text{λz}\text{) = }\text{c}$ |
| Diamond type | $\text{ϕD(}\text{x}\text{, }\text{y}\text{,}\text{z}\text{) = cos(}\text{λx}\text{) cos(}\text{λy}\text{) cos(}\text{λz}\text{)-sin(}\text{λx}\text{) sin(}\text{λy}\text{) sin(}\text{λz}\text{) = }\text{c}$ |
| Primitive type | $\text{ϕP(}\text{x}\text{, }\text{y}\text{,}\text{z}\text{) = cos(}\text{λx}\text{)+cos(}\text{λy}\text{)+cos(}\text{λz}\text{) = }\text{c}$ |
| I-WP type | $\text{ϕ}\text{I}\text{-}\text{WP}\text{(}\text{x}\text{,}\text{y}\text{,}\text{z}\text{)=2[cos(}\text{λx}\text{)cos(}\text{λy}\text{)+}\text{cos}\text{(}\text{λy}\text{)cos(}\text{λz}\text{)+}\text{cos}\text{(}\text{λz}\text{)cos(}\text{λx}\text{)]-}$  $\text{[cos(2}\text{λx}\text{)}\text{+}\text{cos(2}\text{λy}\text{)+cos(2}\text{λz}\text{)]}\text{ = }\text{c}$ |

## Theories and Electromagnetic Simulations

The effective permittivity and transmittance of 3D-printed TPMS structures were measured by using the Floquet theory in CST Studio Suite. The S-parameters were obtained by setting a Floquet port in both the *+z* and *−z* directions and periodic boundary condition. A reliable approach was employed to determine the effective permittivity.^[12-13]^ The scattering parameter has the following relationship with the complex refractive index *n* and the complex impedance *z*.

$$\begin{aligned} z= \pm\sqrt{\frac{{(1+S_{11})}^{2}-{S_{21}}^{2}}{{(1-S_{11})}^{2}-{S_{21}}^{2}}}\#\text{(8)} \end{aligned}$$

$$\begin{aligned} e^{jnk_{0}d}=\frac{S_{21}}{1-S_{11}R_{01}}\#\text{(9)} \end{aligned}$$

$$\begin{aligned} n=\frac{1}{k_{0}d} \left( \mathfrak{I}\left( \ln e^{\mathrm{jn}k_{0}d} \right)+2m\pi-j\mathfrak{K}\left( \ln e^{\mathrm{jn}k_{0}d} \right) \right)\#\text{(10)} \end{aligned}$$

where *k_0_* is the free-space wavenumber, *d* is the thickness of the medium, and *R_01_* is *z*−1*/z*+1. The sign of impedance *z* should satisfy the condition |*e ^jnk^*^0^*^d^* ≤ 1|. In the definition of the complex refractive index *n, m is* branch point, which calculated by introducing the Kramers-Kronig relationship. Finally, the effective permittivity *ϵ_eff_* and effective magnetic permeability *µ_eff_* can be expressed by

$$\begin{aligned} \epsilon_{eff}=\frac{n}{z} , \mu_{eff}=nz\#\text{(11)} \end{aligned}$$

Meanwhile, the electromagnetic transmittance can be defined by

$$\begin{aligned} T={|S_{21}|}^{2}\#\text{(12)} \end{aligned}$$

To reduce the multidirectional scattering of incident microwave, the eigenmode analysis should take the Brillouin zone into account. Within the Brillouin zone, the central location is designated the Γ point. The M point denotes the edge midpoint, and the X point corresponds to the face center.^[14]^ The connection among these points, the periodicity *p* and the wave vector is established by

$$\Gamma=\left( k_{x}p=0, k_{y}p=0 \right)$$

$$X=\left( k_{x}p=\pi, k_{y}p=0 \right)$$

$$\begin{aligned} M=\left( k_{x}p=\pi, k_{y}p=\pi\right)\#\text{(13)} \end{aligned}$$

## Equivalent Specific Strength

Specific strength is an important parameter in material mechanics, which quantifies the stress a material can withstand per unit mass. The corresponding equation is given as follows

$$\begin{aligned} S_{sp}=\frac{\sigma}{\rho}\#\text{(14)} \end{aligned}$$

Where $\sigma$ is the yield strength, and $\rho$ is the equivalent density of lattice structure.

# Supporting Figures


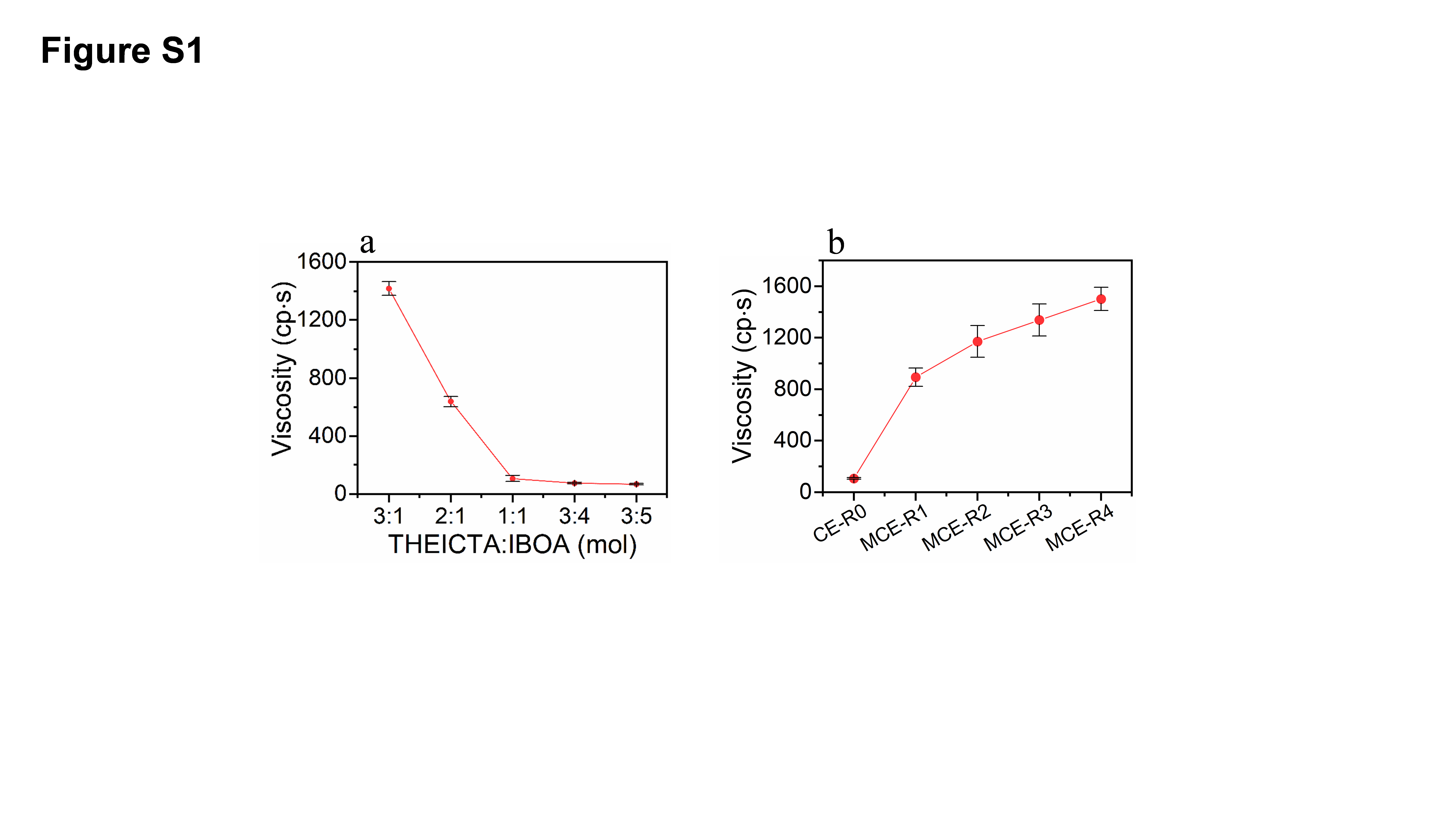


**Figure S1.** a) Viscosity of THEICTA/IBOA at different molar ratios. b) Viscosity of CE-R inks after Michael addition reaction.


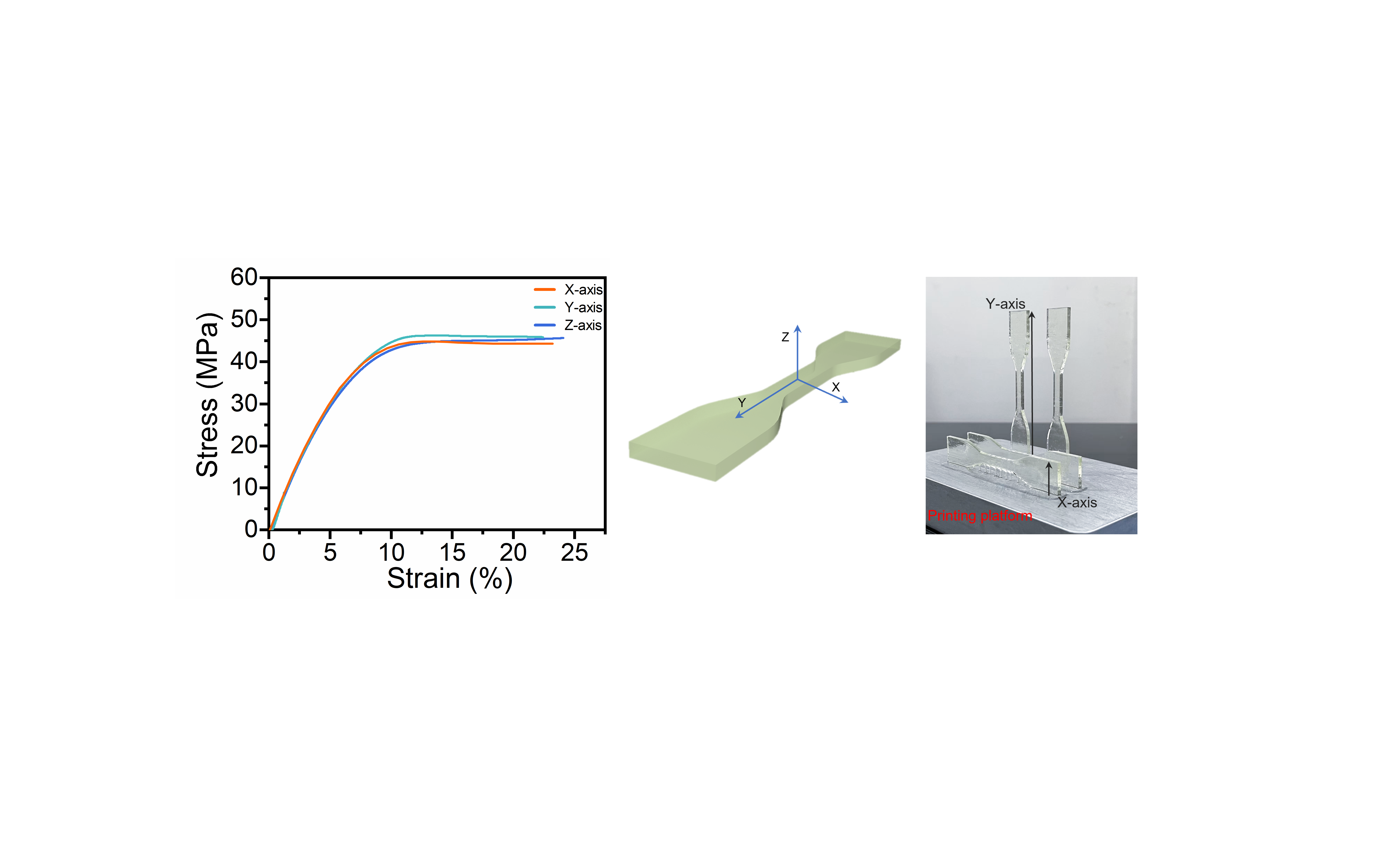


**Figure S2.** Tensile stress–strain curves in the X/Y/Z printing directions and image of the printed specimens.

.


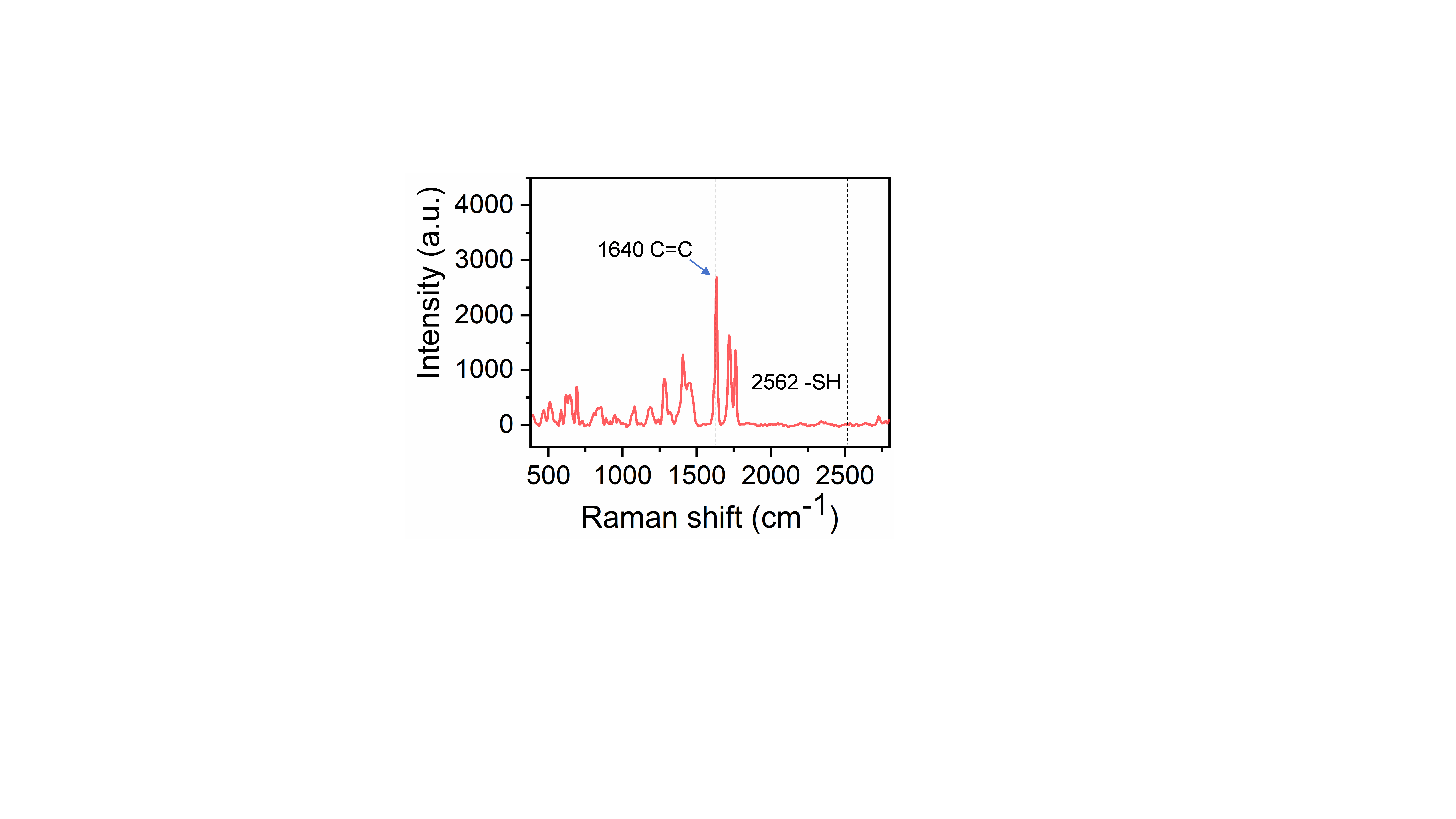


**Figure S3.** Raman spectrum of the MCE-R2 ink.


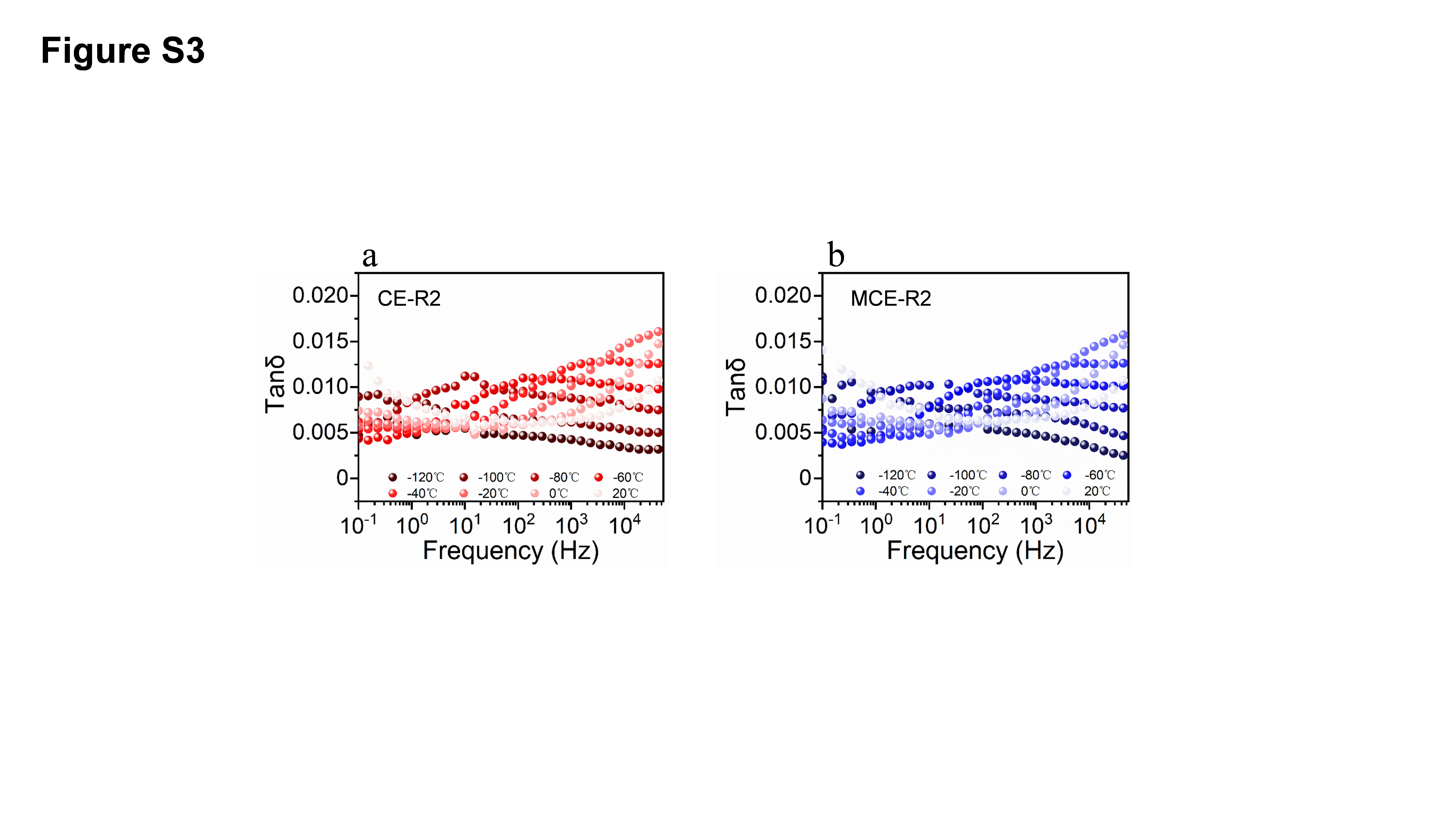


**Figure S4.** Temperature-dependent dielectric loss of a) CE-R2 and b) MCE-R2.


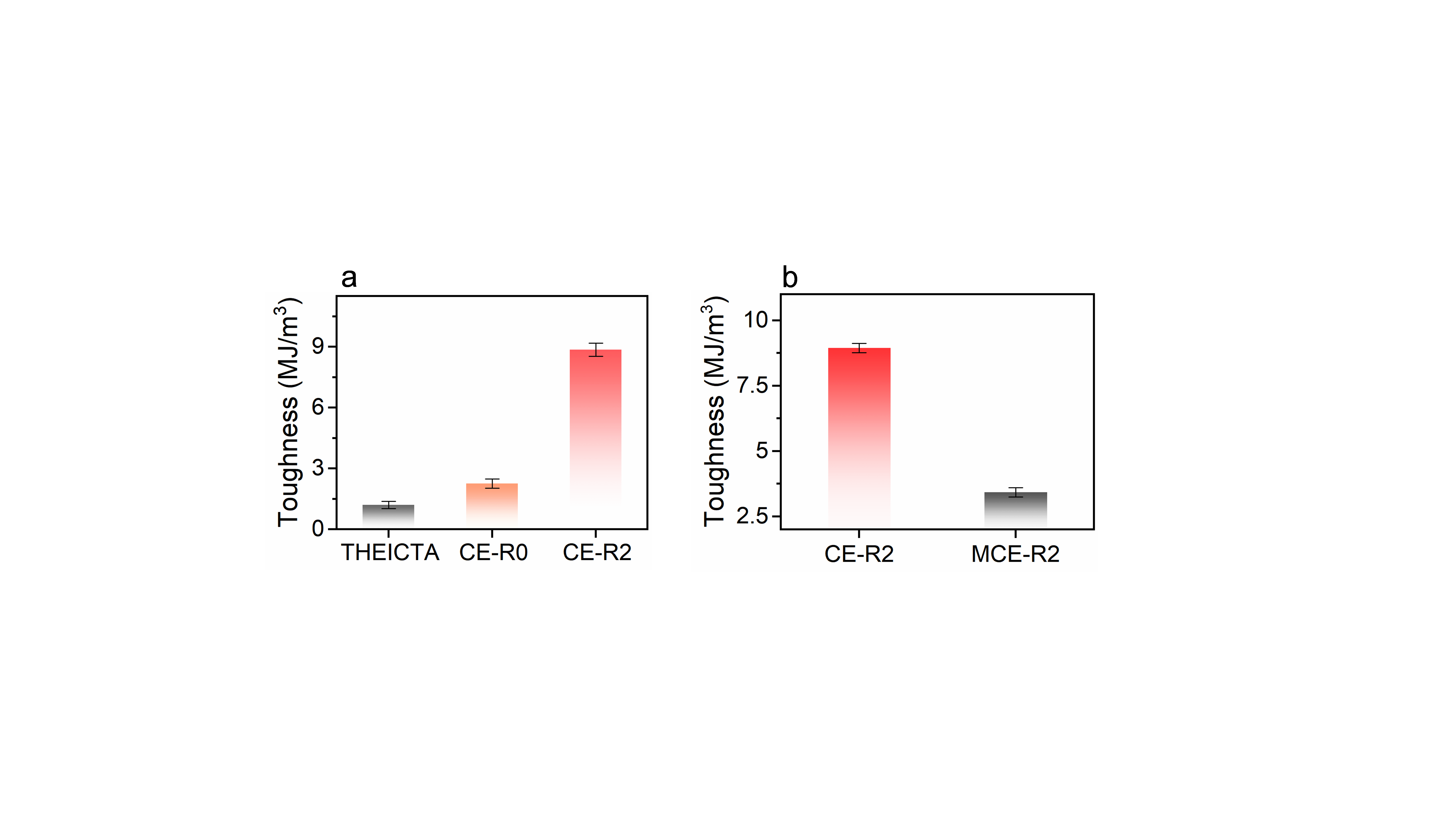


**Figure S5.** a) The tensile toughness comparison of THEICTA, CE-R0 and CE-R2. b) The tensile toughness of CE-R2 and MCE-R2, corresponding to the presence and absence of thiol-acrylate chain transfer behavior, respectively.


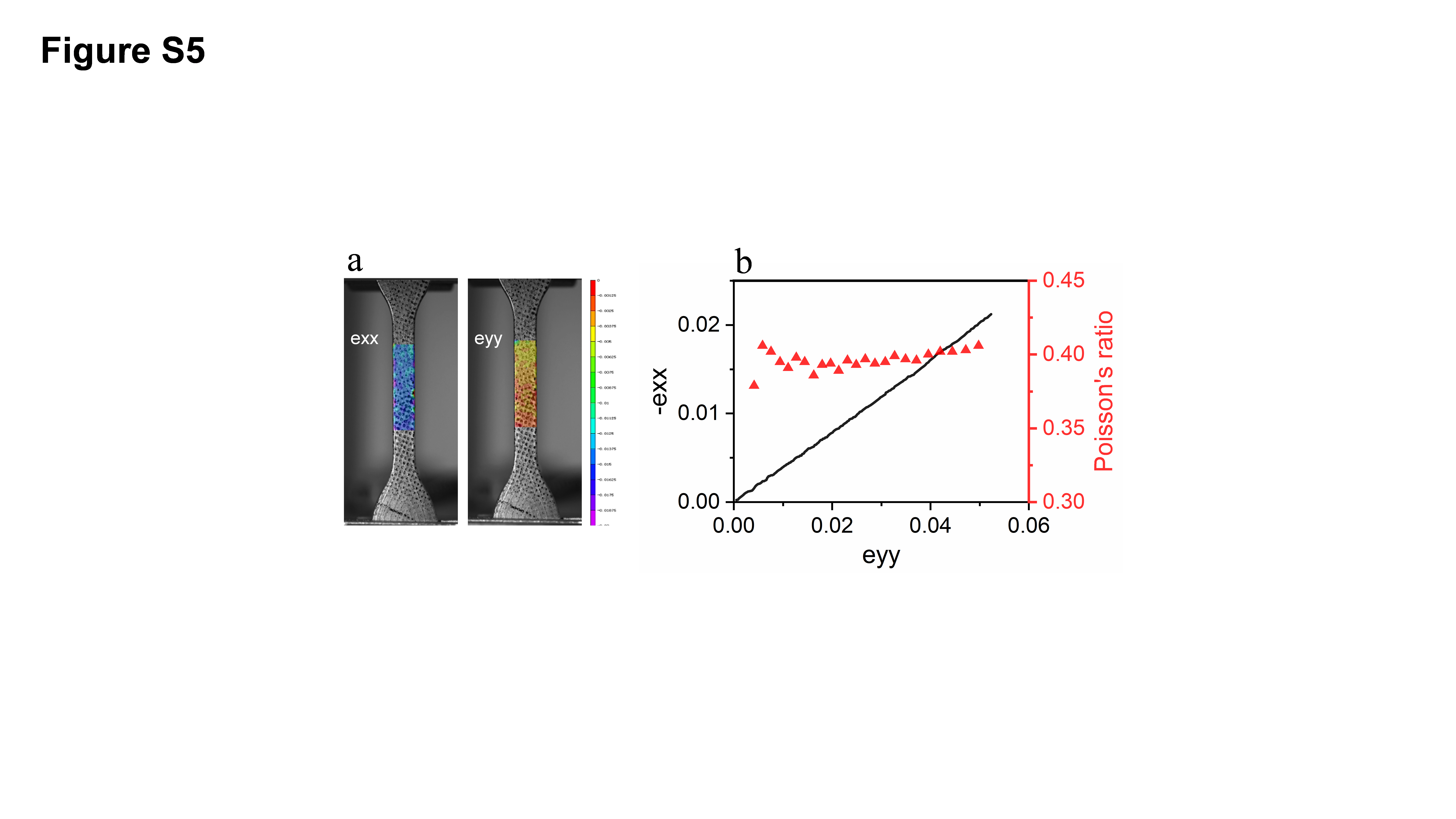


**Figure S6.** a) The tensile specimen for DIC strain measurement (where *exx* represents the transverse strain and *eyy* corresponds to the longitudinal strain). b) The Poisson’s ratio of CE-R2 after UV curing.


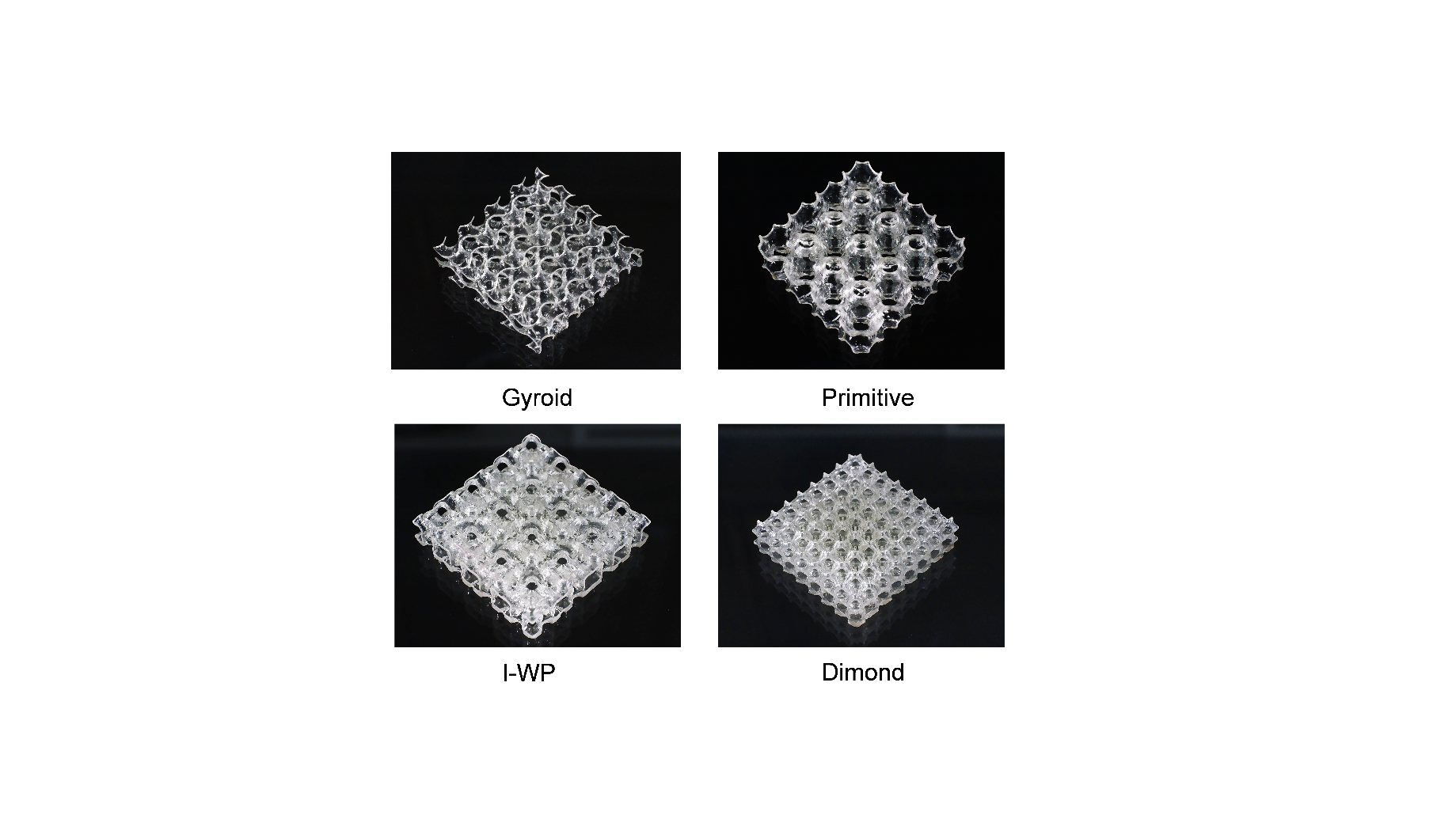


**Figure S7.** 3D printed four kinds of TPMS single-layer lattices (*W* = 0.05 mm) based on CE-R2 ink.


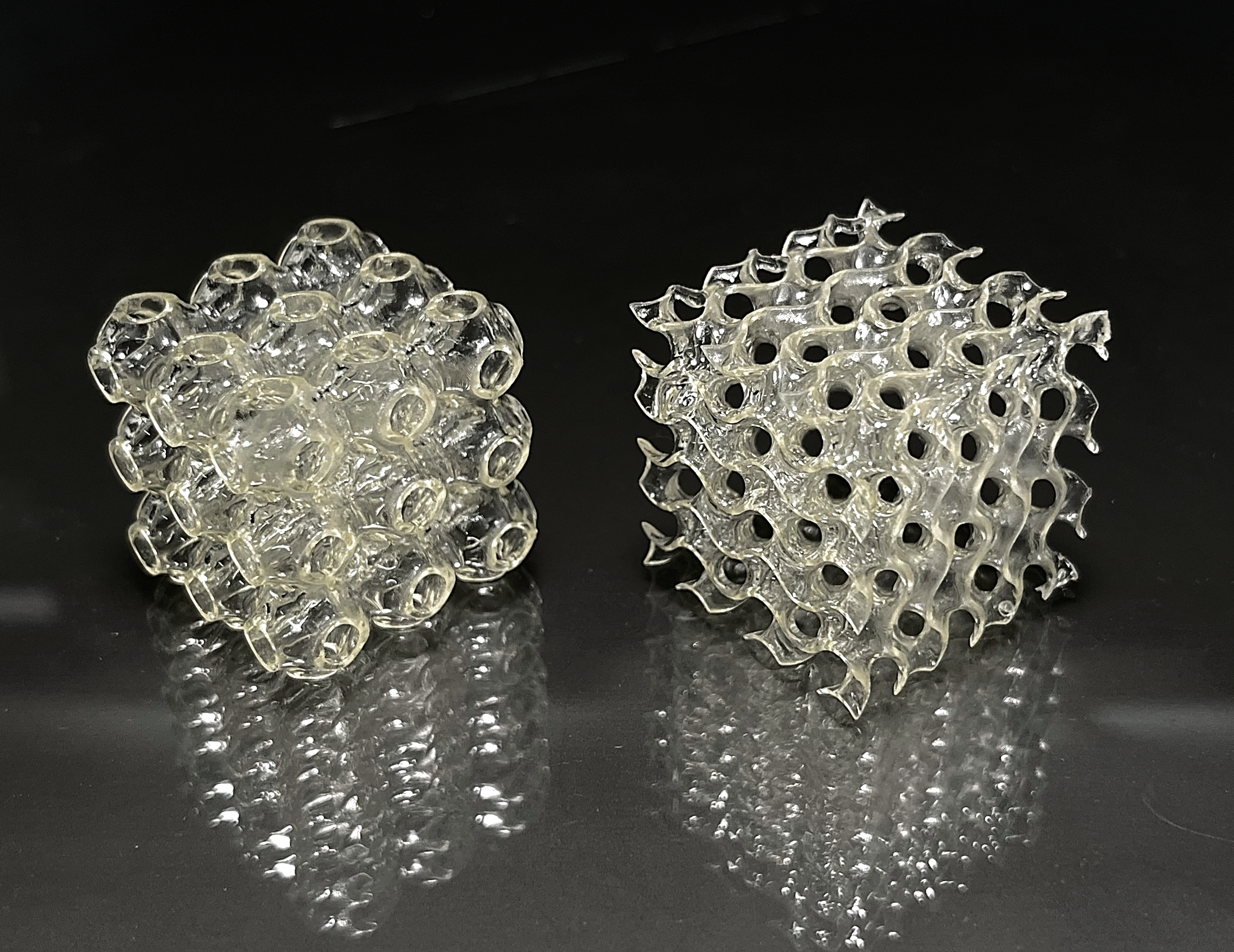


**Figure S8.** 3D-printed 3 cm × 3 cm × 3 cm Gyroid and Primitive lattice structures with 0.5 mm wall thickness.


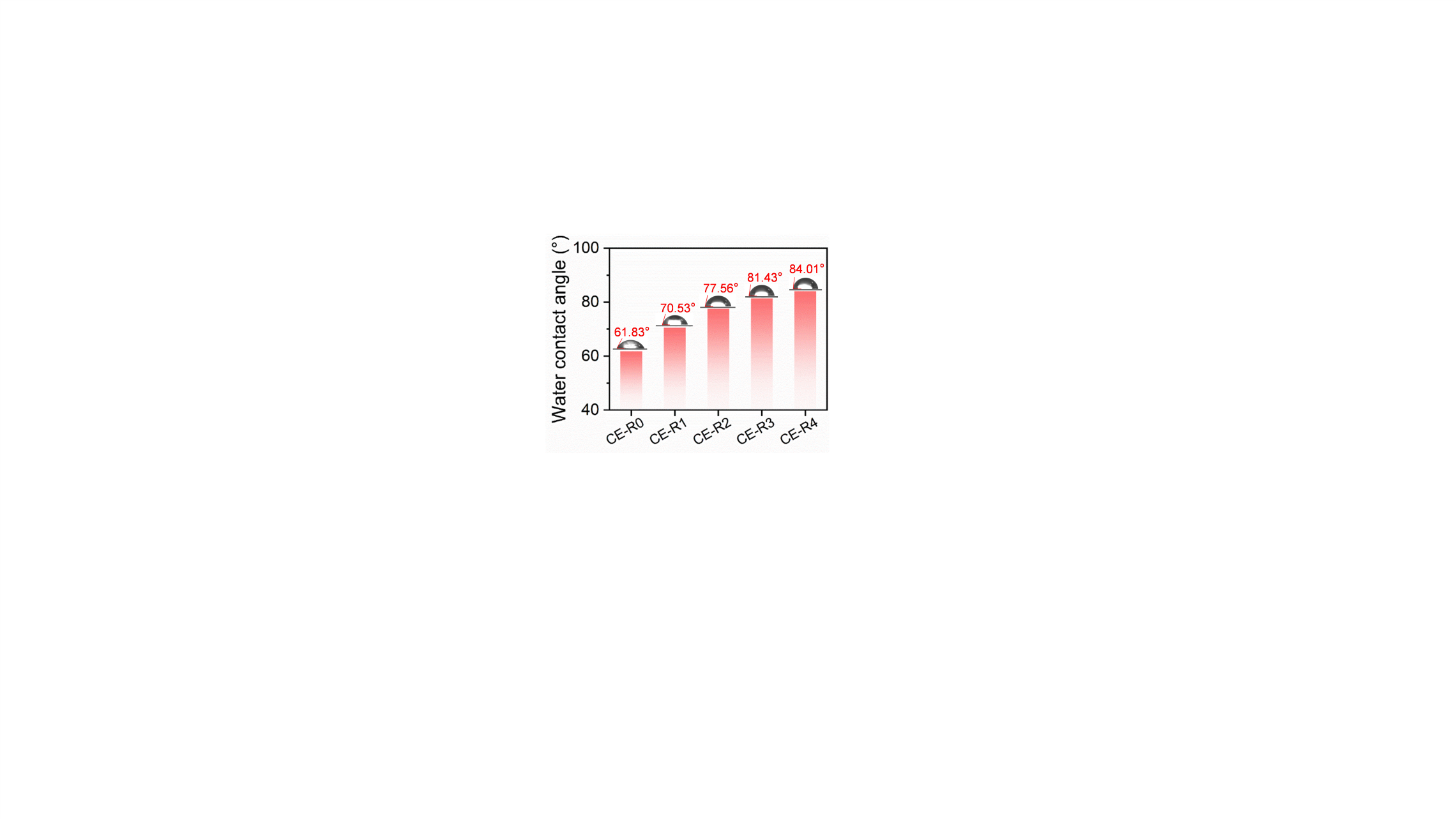


**Figure S9.** Water contact angle of CE-R resins with different PSR content.

# References

[1] Z.-X. Zhou, Y. Li, J. Zhong, Z. Luo, C.-R. Gong, Y.-Q. Zheng, S. Peng, L.-M. Yu, L. Wu, Y. Xu, *ACS Applied Materials & Interfaces* **2020**, 12, 38682.

[2] Z. Lian, Z. Lu, R. Zhao, L. Xiao, L. Hou, *Progress in Organic Coatings* **2024**, 194.

[3] G. Zhang, C. Wang, L. Jiang, Y. Wang, B. Wang, X. Wang, H. Liu, L. Zong, J. Wang, X. Jian, *Polymers* **2023**, 15.

[4] Y.-X. Li, L.-Y. Wu, Y. Yang, Y.-Z. Lu, Z.-Y. Ji, Y.-X. Guo, X.-L. Wang, Q.-S. Pu, *Chinese Journal of Polymer Science* **2024**, 42, 766.

[5] X. Li, J. Shi, C. Kong, C. Li, H. Wang, S. Jiang, Y. Li, *Chemical Engineering Journal* **2024**, 480.

[6] C. Qian, R. Bei, T. Zhu, W. Zheng, S. Liu, Z. Chi, M. P. Aldred, X. Chen, Y. Zhang, J. Xu, *Macromolecules* **2019**, 52, 4601.

[7] C. Hamciuc, E. Hamciuc, M. Olariu, R. Ciobanu, *Polymer International* **2009**, 59, 668.

[8] **!!! INVALID CITATION !!! [4-5]**.

[9] C. M. Costa, A. Reizabal, R. Sabater i Serra, A. A. Balado, L. Pérez-Álvarez, J. L. Gómez Ribelles, J. L. Vilas-Vilela, S. Lanceros-Méndez, *Composites Science and Technology* **2021**, 213.

[10] A. Sieradzki, S. Pawlus, S. N. Tripathy, A. Gągor, M. Ptak, M. Paluch, M. Mączka, *Dalton Transactions* **2017**, 46, 3681.

[11] Y. Li, J. Li, S. Jiang, C. Zhong, C. Zhao, Y. Jiao, J. Shen, H. Chen, M. Ye, J. Zhou, X. Yang, Z. Gou, S. Xu, M. Shen, *Materials Today Bio* **2023**, 20.

[12] Z. Szabó, G.-H. Park, R. Hedge, E.-P. Li, *IEEE Transactions on Microwave Theory and Techniques* **2010**, 58, 2646.

[13] D. M. Pozar, **2005**.

[14] D. Yoon, D.-Y. Na, Y. B. Park, *IEEE Access* **2023**, 11, 140815.
